# Class visualizations and activation atlases for computational pathology

**DOI:** 10.1016/j.xcrm.2026.103054

**Published:** 2026-09-18

**Authors:** Marco Gustav, Fabian Wolf, Christina Glasner, Nic G. Reitsam, Stefan Schulz, Kira Aschenbroich, Bruno Märkl, Sebastian Foersch, Jakob Nikolas Kather

**Affiliations:** 1 Else Kroener Fresenius Center for Digital Health, Faculty of Medicine, TUD Dresden University of Technology, Dresden, Germany; 2 Institute of Pathology, University Medical Center Mainz, Mainz, Germany; 3 Pathology, Faculty of Medicine, University of Augsburg, Augsburg, Germany; 4 Bavarian Cancer Research Center (BZKF), Augsburg, Germany; 5 Department of Medicine I, Faculty of Medicine, TUD Dresden University of Technology, Dresden, Germany; 6 Medical Oncology, National Center for Tumor Diseases (NCT), University Hospital Heidelberg, Heidelberg, Germany; 7 Pathology & Data Analytics, Leeds Institute of Medical Research at St James’s, University of Leeds, Leeds, UK

**Keywords:** computational pathology, deep learning, foundation model, morphology, interpretability, explainable AI, feature visualization, activation atlas, class visualization

## Abstract

The clinical promise of computational pathology increasingly depends on foundation model-based pipelines, yet the morphological concepts encoded by these systems remain poorly understood. We address this gap with a concept-level visualization framework using class visualizations (CVs) and activation atlases (AAs) in a pathology foundation-model setting across colorectal tissue and multi-organ cancer tasks. Four pathologists annotate hematoxylin and eosin-stained image patches as well as generated visualizations, complemented by attribution- and similarity-based metrics. CVs retain class-associated morphology for distinct tissue classes, with reduced separability and higher annotator variability in morphologically overlapping cancer classes. AAs expose layer-dependent organization of encoded concepts, with coherent regions for coarse tissue and cancer groupings and increasing dispersion and overlap at finer label granularities. Together, these findings show that concept-level visualization makes representations learned by pathology foundation models inspectable across diagnostic difficulty levels, with feature separation decreasing and ambiguity increasing as morphological complexity and expert disagreement rise.

## Highlights

• Concept-level explainability exposes morphology encoded by a pathology AI model
• Class visualizations (CVs) reveal prototypical histopathological features
• Activation atlases (AAs) map tissue subclass organization and overlap
• CVs and AAs reflect task complexity and pathologist ambiguity

## Graphical abstract

**Figure:**
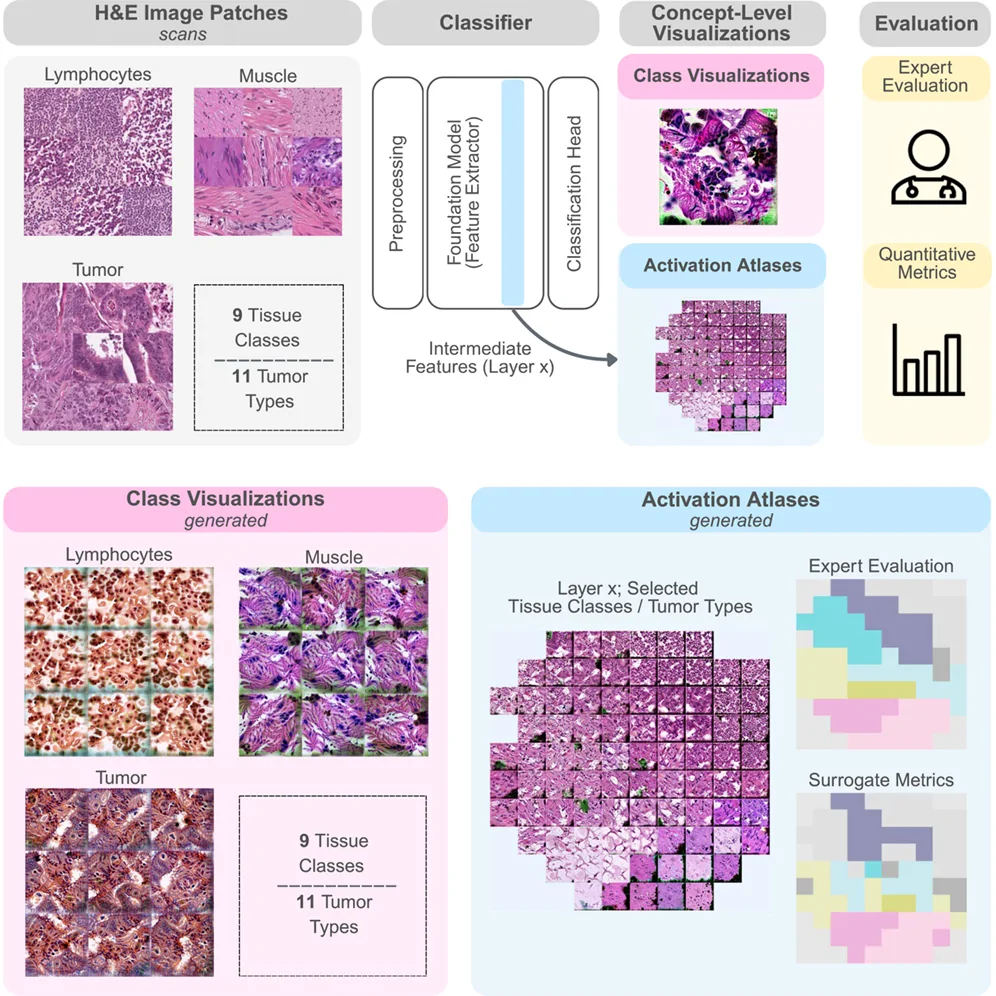

## Teaser

Gustav et al. introduce a concept-level explainability framework that exposes the morphology encoded by a pathology foundation model. The approach reveals prototypical histopathological features and maps the organization and overlap of tissue and cancer concepts, demonstrating that internal model representations reflect both task complexity and pathologist ambiguity.

## Introduction

The expansion of digital pathology and cytology workflows has increased the use of digital solutions and artificial intelligence across diagnostic, educational, research, and laboratory settings.^1^^,^^2^^,^^3^^,^^4^ Within computational pathology, deep learning (DL) models are increasingly used to infer clinically relevant patient-associated features from hematoxylin and eosin (H&E)-stained whole-slide images (WSIs). Weakly supervised learning strategies allow models to learn from slide-level labels without exhaustive region annotations, supporting prediction of molecular biomarkers,^5^^,^^6^^,^^7^^,^^8^^,^^9^^,^^10^^,^^11^^,^^12^^,^^13^^,^^14^^,^^15^^,^^16^ survival,^16^^,^^17^^,^^18^^,^^19^ and treatment response.^13^^,^^14^^,^^20^^,^^21^^,^^22^^,^^23^ Contemporary pipelines typically extract patch-level feature representations using large pre-trained pathology-specific foundation models and aggregate these representations to obtain slide- or patient-level predictions.^13^^,^^24^^,^^25^^,^^26^ Transformer-based architectures have further advanced this paradigm by modeling contextual interactions across image regions, improving performance across diverse computational pathology tasks.^13^^,^^24^^,^^25^^,^^27^

Despite these advances in predictive modeling, the internal organization of learned representations and the morphological patterns driving model predictions remain poorly understood. In computational pathology, explainable artificial intelligence (XAI) methods are predominantly instance-level and focus on explaining individual predictions (explainability), including saliency maps, gradient-based attribution, patch ranking, and low-dimensional embedding visualizations such as t-distributed stochastic neighbor embedding (t-SNE) or uniform manifold approximation and projection (UMAP).^13^^,^^14^^,^^20^^,^^21^^,^^22^^,^^23^ Although these approaches localize image regions associated with predictions, they do not systematically characterize which morphological features define classes, are shared across classes, or constitute higher-order model-derived concepts. In contrast, representation-level interpretability focuses on how models organize and encode concepts within internal feature spaces rather than on explaining specific decisions.^23^ A systematic analysis of representation structure is, therefore, required to characterize class-specific and shared features at the concept level, which is essential for responsible deployment, as trust in DL-based systems depends on alignment between internal representations and established pathological reasoning.^21^^,^^28^ Moreover, systematic interrogation may uncover prediction-relevant morphological patterns warranting further pathological investigation.

XAI approaches in computer vision can be broadly categorized into generative methods, attribution methods, and feature visualization techniques.^29^^,^^30^^,^^31^ Generative approaches, including generative adversarial networks (GANs) and diffusion models, reconstruct visually realistic images from latent variables and can illustrate aspects of a model’s internal feature space but introduce additional modeling components and complexity.^31^^,^^32^ Attribution methods compute and project prediction-specific importance scores onto input pixels without modifying the model and are widely applied in computational pathology.^29^^,^^33^^,^^34^^,^^35^ However, they remain inherently sample-specific and do not directly reveal class-level or slide-agnostic morphological concepts, nor do they characterize continuity and overlap between entities.

Feature visualization methods offer a complementary, model-centric perspective by directly exposing patterns encoded within trained networks.^29^^,^^36^^,^^37^^,^^38^ Class visualization (CV) generates synthetic inputs that maximize class-specific activations, revealing prototypical patterns associated with individual prediction classes. Activation atlases (AAs), in contrast, organize internal activations into low-dimensional space and reconstruct representative feature patterns via feature inversion.^29^^,^^36^^,^^39^ Particularly in convolutional neural networks (CNNs) trained on non-medical images, AAs have revealed coherent concept organization and transitions between visual features.^29^^,^^36^^,^^37^^,^^38^ In computational histopathology, however, such approaches have only been marginally explored and have not been systematically evaluated for modern transformer-based foundation models.^25^^,^^27^^,^^40^ Because vision transformers (ViTs) encode spatial and contextual information differently from CNNs, interpretability techniques developed for these architectures do not transfer directly.^29^^,^^36^^,^^39^ It remains unknown whether transformer-based pathology models organize morphological concepts into structured internal representations that reflect pathological differences and yield features consistently identifiable by expert pathologists.

To address this, we developed a concept-level visualization framework and applied it to a ViT-based classification model trained on H&E-stained image patches, using UNI,^25^ a widely adopted pathology foundation-model backbone, in a frozen configuration with a task-specific linear classification head (Figure 1). Briefly, we trained task-specific linear classifiers on frozen UNI patch embeddings, generated CVs by optimizing synthetic images toward class logits, and generated layer-wise AAs from internal activation vectors across network depth and classification tasks. Analyses were conducted on nine colorectal tissue classes from the National Center for Tumor Diseases (NCT) colorectal cancer dataset (NCT-CRC-HE-100K)^41^ and on multiple solid cancer classification tasks derived from The Cancer Genome Atlas (TCGA)^42^^,^^43^ (Figure 2; Table S1), spanning increasing levels of label granularity and morphological overlap. We evaluated whether the organization of AAs reflects pathological distinctness and whether morphologically similar entities exhibit increased overlap. To this end, four pathologists independently annotated real and generated images to quantify inter-observer agreement, and we implemented quantitative surrogate metrics, including attribution based, perceptual similarity, and distributional distance measures, to assess concordance with expert interpretation. By relating atlas separability to expert agreement across classification settings, we established a systematic framework to evaluate representation structure across tissue and cancer classification tasks.

**Figure 1:**
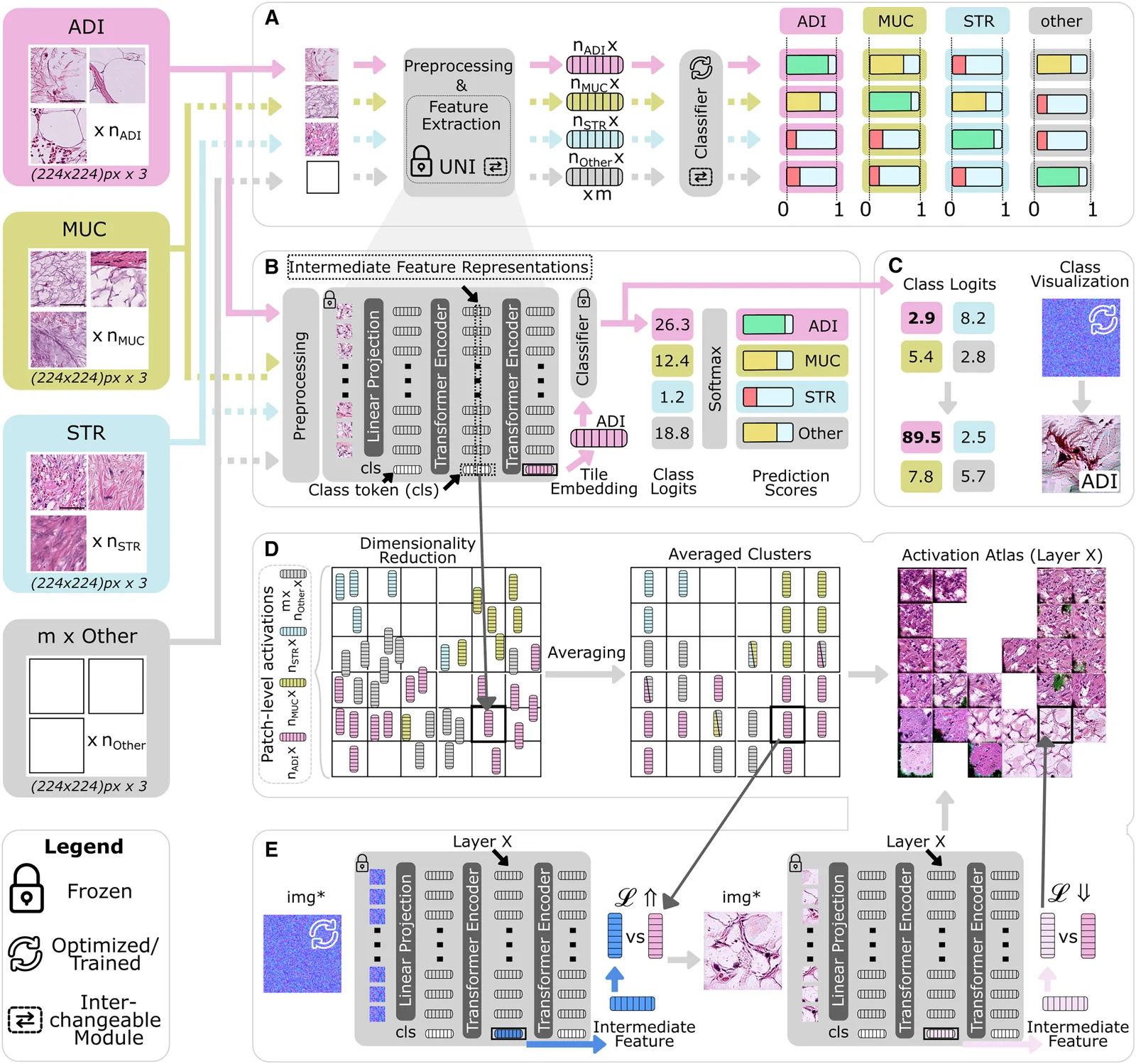
Workflow for feature visualization in transformer-based computational pathology Overview of the proposed framework for analyzing transformer-based representations, including class visualizations and activation atlases for the National Center for Tumor Diseases (NCT) dataset. Scale bars, 50 μm. Icons in the legend were sourced from Canva (https://www.canva.com). (A) Model training. Hematoxylin and eosin (H&E)-stained image patches from multiple tissue (NCT dataset) or cancer classes (TCGA dataset) are used to train a classifier consisting of a frozen vision transformer-based foundation model (UNI^25^) as feature extractor and a trainable linear classification head. The classifier outputs class logits for each patch. The framework is modular, and the backbone, classification head, and training regime can be exchanged for comparative analyses across architectures or fine-tuning strategies. (B) Inner workings and activation capturing. During inference, input patches are processed by the foundation model to produce token-level intermediate feature representations. Activations are captured from selected transformer layers of the trained model. Examples of potential activation capture are indicated by dotted circles. The class token is passed to the linear classification head to produce class logits, which are optionally converted into class probabilities via Softmax for prediction score visualization. In this study, we focused on the class tokens from the respective layers for downstream analyses, as described in the STAR Methods section. (C) Class visualization. Class visualizations are generated by optimizing a synthetic input image to maximize a specific class logit, producing a patch-level image that strongly activates the target class. (D) Dimensionality reduction and averaging. High-dimensional patch-level activations are projected into a two-dimensional embedding space using dimensionality reduction (t-SNE). The embedding space is partitioned into a regular grid, and activations falling into the same grid cell are averaged to obtain representative target feature vectors for downstream visualization. (E) Activation atlases. Feature inversion is applied to reconstruct images from target feature vectors captured in (B). For individual target vectors, optimization minimizes the distance between the activation of the synthetic image and the target activation (illustrated for high- and low-loss [L] cases). For activation atlases, optimized images are arranged according to their positions in the reduced embedding space, yielding a grid that visualizes concept-level morphological patterns learned by the model.

**Figure 2:**
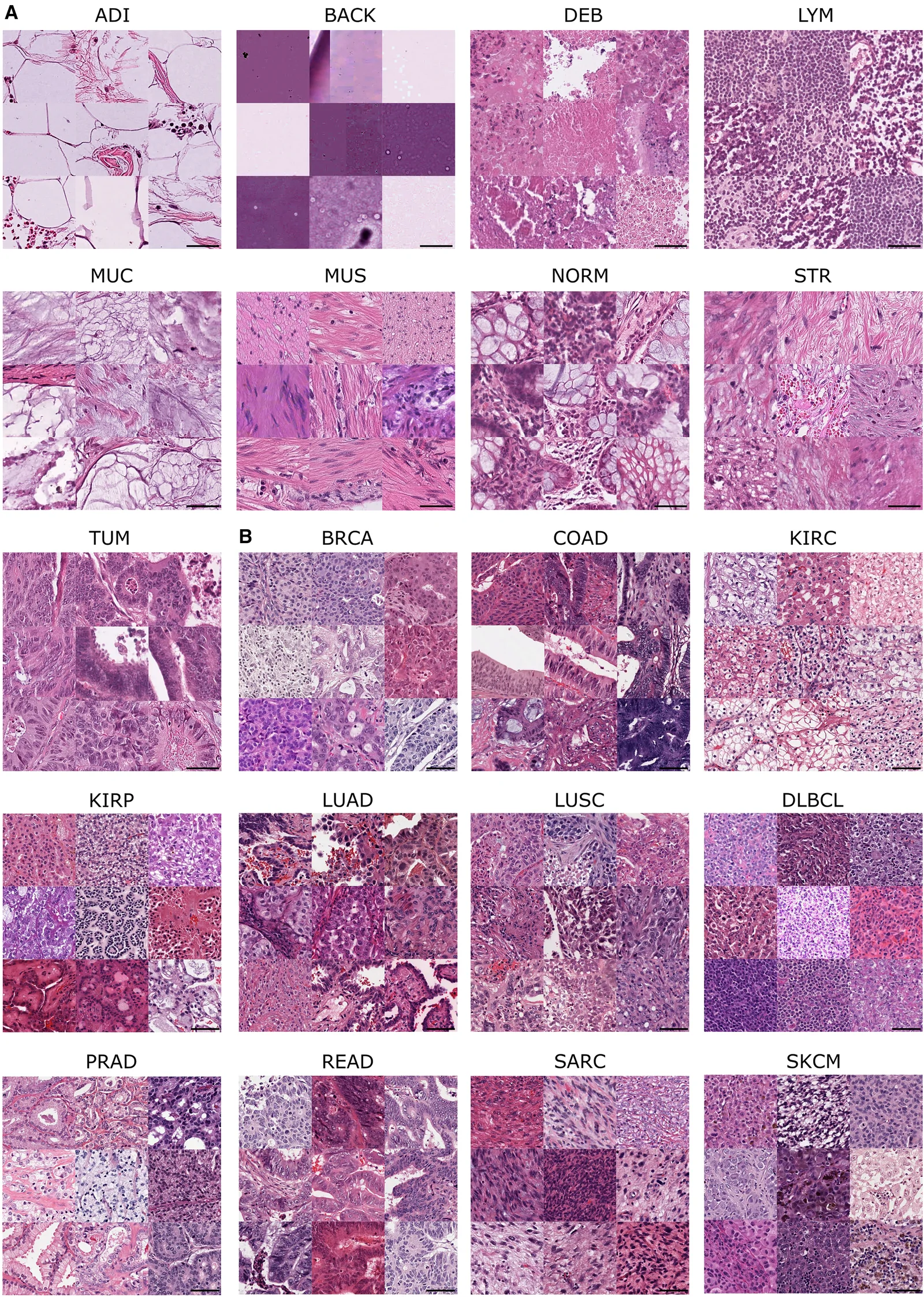
Representative histopathology patches for both datasets Each panel displays example patches illustrating characteristic morphological patterns associated with the corresponding tissue/cancer class as observed in whole-slide images. Scale bars, 50 μm. (A) Representative hematoxylin and eosin (H&E)-stained image patches measuring 224 × 224 pixels at 0.5 μm/pixel, corresponding to a physical area of 112 × 112 μm, for the nine tissue classes used in the National Center for Tumor Diseases (NCT) dataset: adipose tissue (ADI), background (BACK), debris (DEB), lymphocytes (LYM), mucus (MUC), smooth muscle (MUS), normal colon mucosa (NORM), cancer-associated stroma (STR), and colorectal adenocarcinoma epithelium (TUM). (B) Representative H&E-stained image patches measuring 256 × 256 pixels at 0.5 μm/pixel, corresponding to a physical area of 128 × 128 μm, for the 11 cancer classes used from The Cancer Genome Atlas (TCGA) dataset: breast invasive carcinoma (BRCA), colon adenocarcinoma (COAD), kidney renal clear cell carcinoma (KIRC), kidney renal papillary cell carcinoma (KIRP), lung adenocarcinoma (LUAD), lung squamous cell carcinoma (LUSC), lymphoid neoplasm diffuse large B cell lymphoma (DLBCL), prostate adenocarcinoma (PRAD), rectum adenocarcinoma (READ), sarcoma (SARC), and skin cutaneous melanoma (SKCM).

## Results

First, we trained a linear classifier on top of the frozen UNI foundation model and achieved consistently high classification performance across datasets. The area under the receiver operating characteristic curve (AUROC) was 1.00 for NCT and 0.99 for all TCGA tasks; the F1 scores/accuracies were 0.92/0.92 for NCT, 0.83/0.82 for TCGA-5, 0.85/0.85 for TCGA-8, and 0.84/0.86 for TCGA-11. Based on this model, we then evaluated CVs on NCT and TCGA (Figure 3) using performance and agreement metrics to assess class-specific recognizability and inter-annotator consistency, with scan-level annotations as baseline (Figures 4 and 5). Finally, we generated AAs to analyze internal representations and, in the absence of an objective ground truth (GT), assessed inter-annotator agreement and quantitative surrogate metrics (Figures 6 and 7).

**Figure 3:**
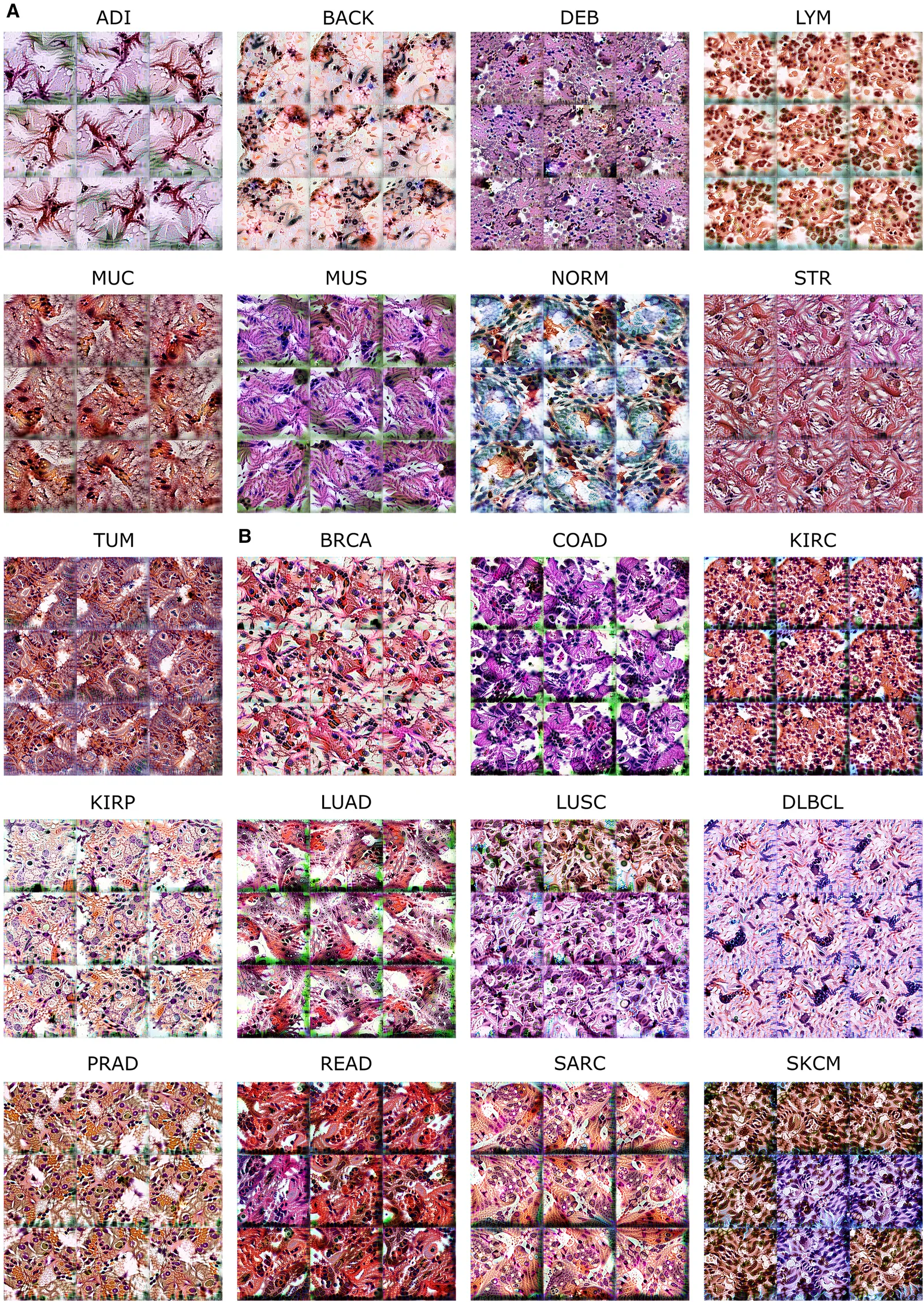
Class visualizations for tissue classes for both datasets For each class, synthetic images were generated by optimizing an input image to maximize the corresponding class logit of a trained vision transformer-based model (UNI^25^), while keeping all model parameters fixed. Each panel displays a grid of independently optimized class visualizations, illustrating morphological patterns that strongly activate the respective class according to the model. The visualizations are instance agnostic and reflect class-level feature representations learned from hematoxylin and eosin (H&E)-stained image patches. Color patterns in class visualizations (CVs) do not represent literal H&E staining. They arise from synthetic image optimization using Fourier-space parameterization followed by RGB reconstruction and may reflect both model-sensitive features and optimization artifacts; CVs are, therefore, interpreted primarily by texture, cellular density, and spatial organization. (A) Shown are class visualizations generated for the nine tissue classes of the National Center for Tumor Diseases (NCT) dataset: adipose tissue (ADI), background (BACK), debris (DEB), lymphocytes (LYM), mucus (MUC), smooth muscle (MUS), normal colon mucosa (NORM), cancer-associated stroma (STR), and colorectal adenocarcinoma epithelium (TUM). The CVs correspond to recognizable and well-characterized histopathological features, representing prototypic histopathological patterns: for adipose tissue, large “empty” fat vacuoles with thin septa (classic fat morphology); for background, pale low-cellularity regions with minimal tissue structure (corresponding to slide background and processing artifacts); for debris, amorphous eosinophilic material with fragmented nuclei and necrotic remnants indicative of tissue breakdown; for lymphocytes, dense small nuclei with high cellularity; for mucus, extracellular mucin pools with sparse cellular elements; for muscle, elongated spindle-shaped cells arranged in bundles with eosinophilic cytoplasm; for normal, relatively organized architecture with epithelial cells; for stroma, fibrous collagen-rich areas; for tumor, crowded pleomorphic epithelial cells forming irregular glandular patterns (characteristic of CRC). (B) Shown are class visualizations generated for 11 cancer classes from The Cancer Genome Atlas (TCGA): breast invasive carcinoma (BRCA), colon adenocarcinoma (COAD), kidney renal clear cell carcinoma (KIRC), kidney renal papillary cell carcinoma (KIRP), lung adenocarcinoma (LUAD), lung squamous cell carcinoma (LUSC), lymphoid neoplasm diffuse large B cell lymphoma (DLBCL), prostate adenocarcinoma (PRAD), rectum adenocarcinoma (READ), sarcoma (SARC), and skin cutaneous melanoma (SKCM). The CVs correspond to prototypic histopathological patterns of the respective cancer types. For most adenocarcinomas (COAD, READ, LUAD, and PRAD), the visualizations share common glandular architectural features reflecting their shared epithelial origin. For BRCA, irregular ductal or lobular arrangements of pleomorphic tumor cells embedded in desmoplastic stroma are prominent. For KIRC, tumor cells appear organized in nested or sheet-like configurations, consistent with the characteristic clear cell architecture, while KIRP visualizations suggest a papillary growth pattern. For LUSC, the visualizations resemble nests or sheets of polygonal squamous cells with features of keratinization. For SARC, spindle-shaped cellular arrangements in fascicular patterns reflect the mesenchymal origin of these tumors. For SKCM, a diffuse growth pattern with occasional melanin deposition is apparent. For DLBCL, diffuse sheets of large lymphoid cells with prominent nuclei are visible.

**Figure 4:**
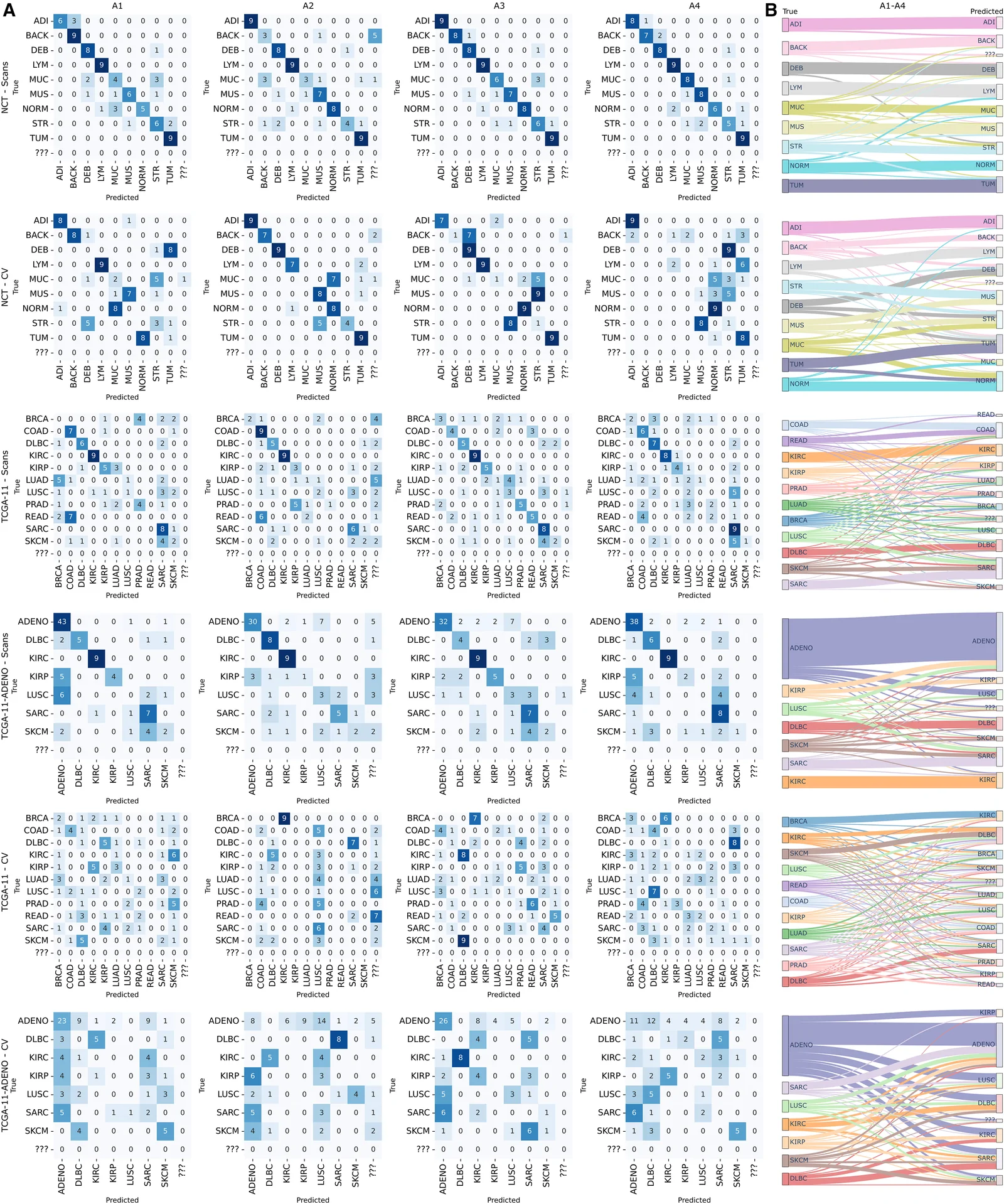
Pathologist annotation patterns for scan images and class visualizations across tasks We evaluated pathologist annotations for real scan images and model-derived class visualizations (CVs) across colorectal tissue classification (National Center for Tumor Diseases [NCT] dataset) and multi-cancer classification settings derived from The Cancer Genome Atlas (TCGA), including analyses at different levels of label granularity. The NCT task comprises nine colorectal tissue classes: adipose tissue (ADI), background (BACK), debris (DEB), lymphocytes (LYM), mucus (MUC), smooth muscle (MUS), normal colon mucosa (NORM), cancer-associated stroma (STR), and colorectal adenocarcinoma epithelium (TUM). TCGA-based settings comprise 11 solid tumor entities: breast invasive carcinoma (BRCA), colon adenocarcinoma (COAD), kidney renal clear cell carcinoma (KIRC), kidney renal papillary cell carcinoma (KIRP), lung adenocarcinoma (LUAD), lung squamous cell carcinoma (LUSC), lymphoid neoplasm diffuse large B cell lymphoma (DLBCL), prostate adenocarcinoma (PRAD), rectum adenocarcinoma (READ), sarcoma (SARC), and skin cutaneous melanoma (SKCM). An additional adenocarcinoma-level grouping (ADENO) collapses morphologically related adenocarcinoma entities into a single class. An additional category (“???”) denotes uncertain assignments. (A) Row-normalized confusion matrices summarizing individual pathologist annotations (A1–A4) for scanned images and CVs. Rows denote reference labels (ground truth for scans; optimization objective class for CVs), and columns denote annotated labels. Color intensity represents the proportion of samples within each reference class assigned to a given label; overlaid numbers indicate raw annotation counts. (B) Sankey diagrams showing the aggregated correspondence between reference labels (left) and pathologist-assigned labels (right), based on annotations summed across all pathologists. Ribbon widths are proportional to the number of annotated samples per label pair and depict class-wise agreement, systematic confusions, and label uncertainty.

**Figure 5:**
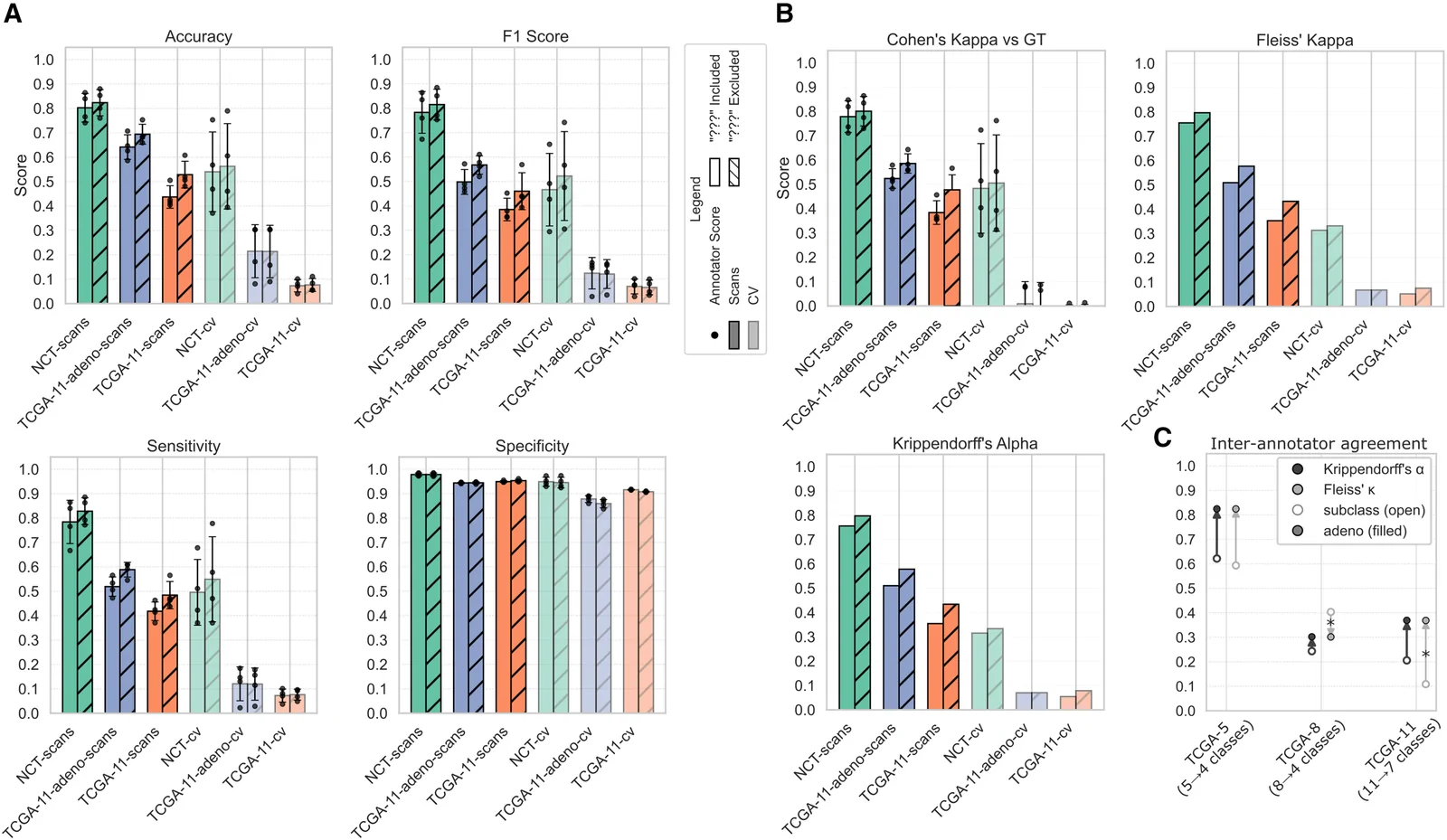
Annotation performance and agreement metrics for class visualizations and activation atlases across datasets Summary of pathologist annotation performance and agreement for real scan images and class visualizations (CVs) across the National Center for Tumor Diseases (NCT) tissue dataset and The Cancer Genome Atlas (TCGA)-derived classification tasks. Metrics are shown separately for real scan images (scans) and CVs and for different label granularities, including the NCT tissue classification task, the TCGA-11 cancer classification task, and an adenocarcinoma-level grouping (TCGA-11-adeno), in which multiple adenocarcinoma entities are collapsed into a single class (ADENO). Hatched bars indicate analyses excluding uncertain annotations (“???”), whereas solid bars indicate analyses including uncertain labels as an explicit category. Metrics are reported to characterize annotation consistency and ambiguity rather than diagnostic accuracy or explanation correctness. All 42 scan-CV comparisons were statistically significant: scans exceeded CVs throughout, with 95% bootstrap confidence intervals excluding zero. Significance was assessed by two-sided stratified item-level permutation testing with Holm correction across all comparisons; all adjusted *p* values were at the permutation-resolution limit and are reported as upper bounds (Holm-adjusted *p* < 0.005). Full statistics are provided in Table S2. (A) Descriptive performance metrics including accuracy, F1 score, sensitivity, and specificity, computed with respect to reference labels. Bar heights indicate the mean of annotator-specific scores for each task and metric, error bars denote the standard deviation across annotators, and overlaid points represent individual annotator scores. (B) Agreement metrics include Cohen’s κ (agreement between individual annotators and reference labels), Fleiss’ κ (overall agreement among multiple annotators), and Krippendorff’s α, which accounts for missing labels. For Cohen’s κ versus ground truth, bar heights indicate the mean across annotators, error bars denote the standard deviation, and overlaid points represent individual annotators. For Fleiss’ κ and Krippendorff’s α, bars indicate a single group-level agreement value without error bars. (C) Cross-task summary of inter-annotator agreement for activation atlas annotations across TCGA tasks. Fleiss’ κ and Krippendorff’s α are shown for TCGA-5, TCGA-8, and TCGA-11 at subclass level and after adenocarcinoma-level label collapsing. Open symbols indicate subclass-level analyses, and filled symbols indicate adenocarcinoma-collapsed analyses. Agreement was generally higher after label collapsing, consistent with reduced ambiguity at coarser granularity. Detailed task-specific activation atlas annotations, label maps, class coverage, and metric-alignment analyses are provided in Figures S6–S17. An asterisk (∗) indicates that the Fleiss’ κ subclass estimate is based on <50% complete atlas tiles because tiles with uncertain annotations were excluded; this estimate should, therefore, be interpreted cautiously.

**Figure 6:**
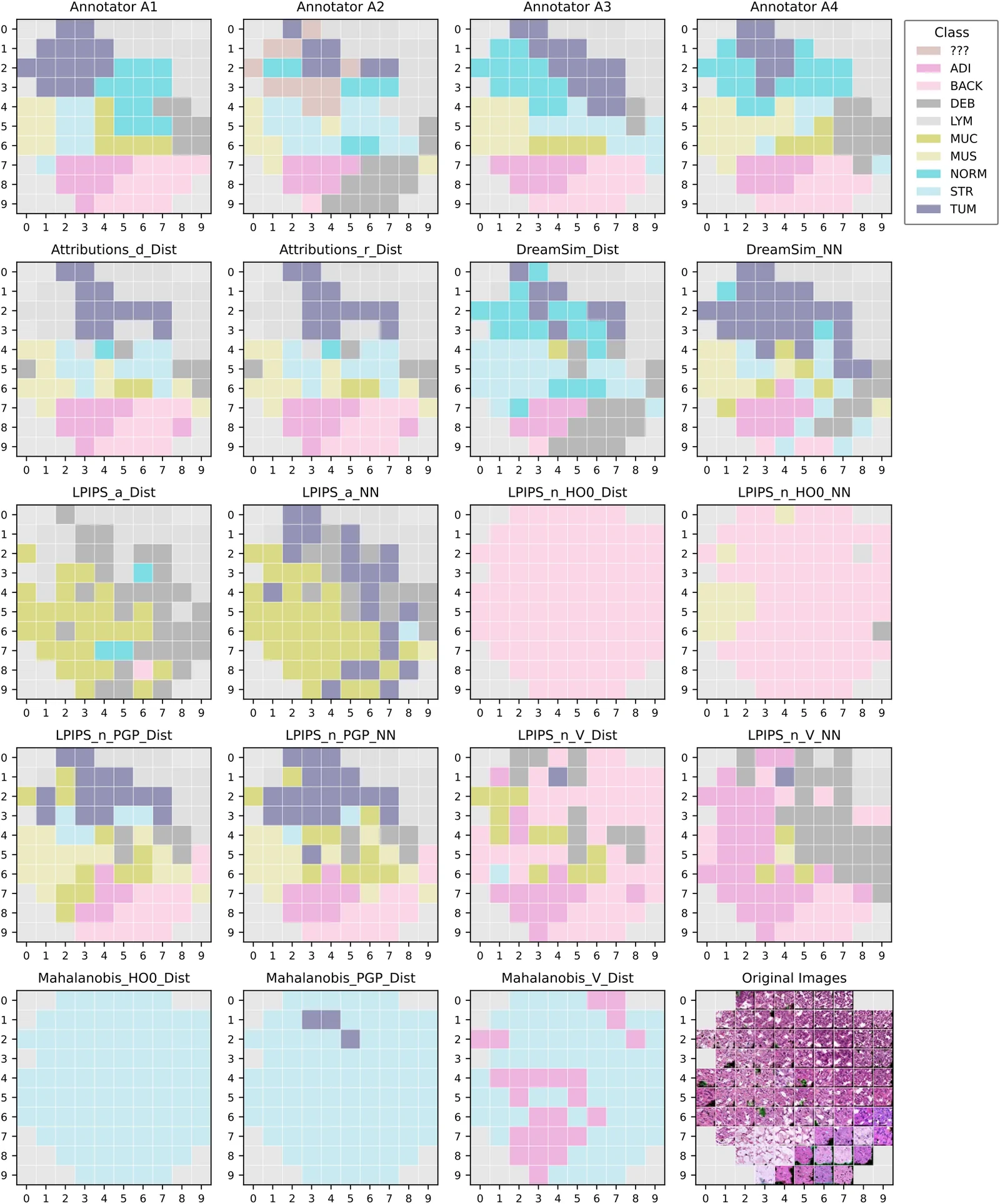
Label maps for activation atlas cells in the NCT tissue dataset Cell-wise label maps for an activation atlas derived from the NCT dataset. Each grid shows the same activation atlas layout, in which each cell corresponds to an aggregated region in the reduced activation embedding space. Cell colors indicate the class label assigned to each activation atlas cell. See also Figures S1–S6, S8, S10, S12, S14, and S16. Top row: independent annotations by four pathologists (annotators A1–A4), assigning one of the nine NCT tissue classes: adipose tissue (ADI), background (BACK), debris (DEB), lymphocytes (LYM), mucus (MUC), smooth muscle (MUS), normal colon mucosa (NORM), cancer-associated stroma (STR), and colorectal adenocarcinoma epithelium (TUM), or an explicit label for uncertainty (“???”). Middle rows: corresponding atlas label maps obtained using quantitative surrogate assignment methods. Attribution-based methods assign labels based on class attributions aggregated over atlas cells, using either distance-based aggregation (_Dist; assignment based on minimal attribution distance) or nearest-neighbor-based aggregation (_NN; assignment based on the closest individual activations). Perceptual similarity-based methods assign each atlas cell to the closest class using either DreamSim or Learned Perceptual Image Patch Similarity (LPIPS), again using distance-based (_Dist) or nearest-neighbor (_NN) strategies. LPIPS variants differ by the feature extractor used, indicated by suffixes _a (AlexNet), _H00 (H-optimus-0), _PGP (Prov-GigaPath), and _V (vision transformer, UNI). Bottom left: Mahalanobis distance-based methods assign labels based on class-conditional feature distributions computed in the corresponding representation spaces. Bottom right: generated image patches associated with each activation atlas cell, illustrating the underlying morphological content contributing to the aggregated activations.

**Figure 7:**
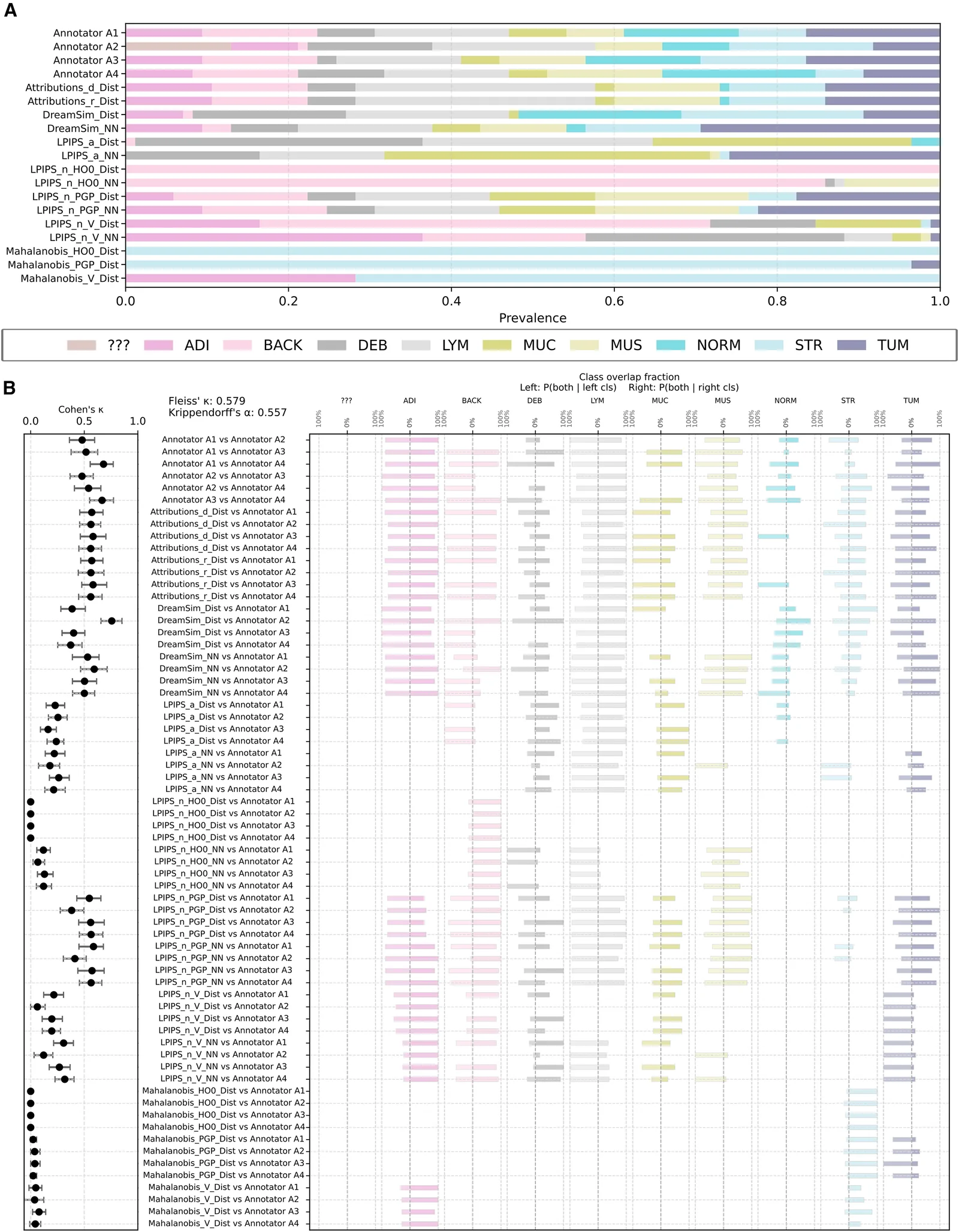
Agreement and class coverage for activation atlas annotations in the NCT tissue task Agreement analysis and class coverage for activation atlas cell annotations derived from the NCT tissue classification task. See also Figures S7, S9, S11, S13, S15, and S17. (A) Class coverage showing the distribution of class assignments across activation atlas cells for each pathologist (annotators A1–A4) and for each metric-based label assignment method. Bars indicate the proportion of atlas cells assigned to each NCT tissue class: adipose tissue (ADI), background (BACK), debris (DEB), lymphocytes (LYM), mucus (MUC), smooth muscle (MUS), normal colon mucosa (NORM), cancer-associated stroma (STR), and colorectal adenocarcinoma epithelium (TUM). (B) Agreement metrics and class-wise overlap. Pairwise agreement between annotators and between annotators and metric-based assignments, computed excluding uncertain labels (“???”) to ensure comparability of categorical agreement. Left: Cohen’s κ values summarizing pairwise agreement, with points indicating mean κ across atlas cells and horizontal error bars denoting uncertainty estimated by bootstrap resampling. Overall inter-annotator agreement is summarized by Fleiss’ κ and Krippendorff’s α (top). Right: class-wise overlap fractions indicating, for each tissue class, the fraction of atlas cells that received the same label from both compared sources. Bars on the left show, for each tissue class, how often atlas cells with that reference label are assigned the same class by the comparator. Bars on the right show, for each assigned class, how often the assigned cells originate from the corresponding reference label. Together, these measures capture asymmetric agreement and class-specific ambiguity across expert annotations and quantitative surrogate metrics.

### CVs preserve detectability of multiple tissue classes

On NCT, scan annotations (Figure 2A) showed high inter-annotator agreement and clear separability for most tissue classes, including adipose (ADI), lymphocytes (LYM), colorectal adenocarcinoma epithelium (TUM), smooth muscle (MUS), normal colon mucosa (NORM), and debris (DEB), with minor discrepancies (e.g., mucus [MUC] versus cancer-associated stroma [STR]) and infrequent use of the uncertain label (Figure 4). CVs largely preserved class-specific patterns (Figure 3A) and maintained consistent detectability for several classes (ADI, LYM, NORM, TUM), capturing recognizable morphologies such as large lipid vacuoles in adipose tissue, dense lymphocytic infiltrates, and crowded pleomorphic glands in tumor epithelium; however, separability decreased and inter-annotator variability increased, particularly between STR and MUS (Figure 4). Thus, NCT CVs preserved class-discriminative information for multiple morphologically distinct tissue classes, although agreement and separability were reduced compared with scan images (Figure 5).

### CVs show reduced separability and high annotator variability across multiple solid cancer classes

In contrast, TCGA-11 CVs showed substantially reduced separability and stronger annotator-dependent variability (Figure 3B). CVs still reflected characteristic histopathological patterns across selected entities, including shared glandular architecture among adenocarcinomas from different organs (colon adenocarcinoma [COAD], lung adenocarcinoma [LUAD], and prostate adenocarcinoma [PRAD]), clear cell nesting in kidney renal clear cell carcinoma (KIRC), spindle-cell fascicles in sarcoma (SARC), and diffuse lymphoid infiltrates in lymphoid neoplasm diffuse large B cell lymphoma (DLBCL). However, discrimination was reduced among morphologically overlapping classes, with frequent cross-assignments at the adenocarcinoma (ADENO) level (e.g., ADENO as lung squamous cell carcinoma [LUSC], DLBCL, SARC, or KIRC; SARC as ADENO), while ADENO and skin cutaneous melanoma (SKCM) retained relatively higher consistency (Figure 4). At the subclass level, ambiguity further increased, with broadly distributed assignments and recurrent misclassifications (e.g., breast invasive carcinoma [BRCA] as KIRC, DLBCL as SARC, and KIRC or SKCM as DLBCL), resulting in reduced performance and minimal consensus for fine-grained CV interpretation (Figures 4 and 5). Together, these results indicate that CVs retained class-discriminative information for selected classes but were consistently less interpretable than real scans, with significantly lower performance across all datasets and uncertainty settings (42 comparisons; Δ = 0.03–0.59; Holm-adjusted *p* < 0.005; Figure 5; Table S2). This reduction was most pronounced with increasing dataset complexity, morphological overlap, and label granularity, consistent with the greater difficulty of distinguishing heterogeneous tumor entities compared with well-defined tissue classes.

### AAs capture consistent tissue-level concepts in the NCT dataset

Following the instance-level CV analysis, we next examined AAs to evaluate concept-level representations beyond individual logits. AA cells were independently annotated by four pathologists and compared with metric-derived labels; in the absence of an objective semantic GT, expert annotations served as reference. As an exploratory layer-wise analysis, we examined how visualized patterns evolved across the UNI network depth. NCT AAs and embeddings showed increasing structural detail and class differentiation across transformer layers (Figures S1–S5). Early layers encoded low-level morphology with limited separability, intermediate layers exhibited coherent concept organization, and deeper layers produced more detailed but increasingly specialized representations. We, therefore, selected layer 14/24 as a balance between separability and over-specialization for subsequent analyses.

For NCT, inter-annotator agreement was moderate (Fleiss’ κ = 0.58; Krippendorff’s α = 0.56) with comparable pairwise Cohen’s κ across annotator pairs, but varied across classes and cells, which often received heterogeneous labels (Figures 6 and 7). Attribution-based assignments achieved agreement within the inter-annotator range and reproduced class prevalence closely matching expert annotations without mode collapse. Among perceptual metrics, nearest-neighbor (NN)-based DreamSim and learned perceptual image patch similarity (LPIPS) outperformed distance-based variants; DreamSim reached higher κ than LPIPS but showed greater annotator-dependent variability (Figure 7), while LPIPS with Prov-GigaPath overlapped attribution-level agreement in selected comparisons. DreamSim distributed prevalence across more classes, whereas LPIPS concentrated assignments depending on the feature extractor (Figure 6). In contrast, Mahalanobis distance (MD) showed near-zero agreement across extractors and collapsed assignments to few classes, with minimal overlap with expert labels.

### Annotator agreement and metric alignment in AAs vary with label complexity in TCGA cancer classes

Next, we analyzed AAs across TCGA class sets to assess the effect of label granularity (Figures 5C and S6–S17). Across TCGA tasks, agreement decreased with increasing class complexity, morphological overlap, and label granularity. Analyses using broader adenocarcinoma-level groupings generally showed higher agreement and more coherent atlas regions, whereas subclass-level analyses showed increased dispersion, heterogeneous annotator assignments, and reduced metric alignment. Attribution-based labels generally remained within the inter-annotator agreement range at lower label granularity and distributed assignments across classes without strong dominance. Among perceptual metrics, NN-DreamSim and NN-LPIPS variants (e.g., Prov-GigaPath) performed better than distance-based approaches, although rankings varied by class set and feature extractor. DreamSim tended to concentrate assignments on dominant classes, whereas LPIPS distributed them more broadly. MD again showed near-zero agreement and collapsed assignments to few classes.

### Exploratory visualizations of normal adjacent and tumor tissue capture organ-specific and malignancy-associated morphology

As an exploratory extension, we applied the visualization framework to matched normal adjacent and tumor regions from selected TCGA cohorts (Figure S18). CVs of normal adjacent tissue showed recognizable organ-specific non-neoplastic architecture, such as colonic crypts with goblet cells in COAD, whereas tumor CVs frequently showed malignancy-associated morphology, including hyperchromasia and nuclear pleomorphism. AAs showed clearer separation between normal adjacent and tumor tissue in entities with distinct routine histopathological morphology, such as COAD, whereas hepatocellular carcinoma (LIHC) showed less-distinct clustering, consistent with the more subtle tumor-background separation in this setting.

Overall, AAs captured tissue- and cancer-level concepts at coarse label resolutions, with agreement and metric alignment comparable to inter-observer variability; both declined with increasing label granularity, paralleling reduced expert consensus in real images and the patterns observed for instance-level CVs, indicating sensitivity to dataset complexity.

## Discussion

Concept-level feature visualization demonstrated that transformer-based pathology models organize morphological information into structured latent representations. CVs and AAs did not provide mechanistic explanations of individual predictions but revealed how concepts and their relationships were encoded. Coarse tissue categories yielded coherent, well-separated patterns, whereas increasing label granularity resulted in greater dispersion and overlap, indicating that separability scaled with morphological complexity and that transformer representations preserved intrinsic pathological structure rather than imposing artificial boundaries. Layer-wise analyses showed progressively increased structural detail and class differentiation with depth, consistent with prior CNN findings.^36^^,^^44^

At the level of individual classes, CVs recapitulated prototypic histomorphological features, such as dense aggregates of small hyperchromatic nuclei for lymphocytes and optically empty vacuoles within thin septa for adipose tissue. Reduced separability between STR and MUS in the NCT dataset, accompanied by increased inter-annotator variability, likely reflected their overlapping eosinophilic staining and shared spindle-cell morphology in colorectal tissue (e.g., collagen-rich stroma versus smooth muscle bundles). In the TCGA setting, CVs could be used to identify hallmarks of healthy tissue and the respective malignant counterpart. Comparing different tumor types, CVs likewise captured recognizable patterns in selected entities, but their substantially lower agreement compared with scans underscored that they should not be interpreted as diagnostic images. Rather, CVs are concept-level visualizations designed to expose model- and task-associated morphological patterns, whose recognizability and ambiguity can be assessed by experts. AAs further revealed continuity between related entities. In the NCT dataset, internal representations were compact and distinct, whereas morphologically overlapping TCGA cancer classes exhibited increased overlap and annotator-dependent ambiguity. Reduced apparent atlas separability paralleled lower expert agreement on real images, suggesting covariation with pathological complexity and expert uncertainty; however, embedding-dependent atlas topology should not be interpreted as a quantitative measure of global representational distance. The exploratory normal-adjacent tissue-versus-tumor analysis further illustrates how the framework may inspire use cases by revealing whether models encode organ-specific context, malignancy-associated morphology, and transitions between them.

Concept-level visualization complements attribution methods that localize relevance within individual images^21^^,^^22^ and generative approaches relying on auxiliary models.^31^^,^^32^ CVs identify patterns that maximize specific logits, whereas AAs expose density, continuity, and overlap of representations across layers, which is particularly relevant for transformer architectures, where distributed representations and token interactions complicate interpretation through localized relevance maps alone.^29^^,^^36^^,^^40^ The dependence on dataset complexity further indicates that representation structure is task specific and not uniformly separable across granularities.

Quantitative surrogate metrics showed variable alignment with expert interpretation; attribution-based assignments achieved agreement comparable to inter-pathologist variability in settings with limited morphological overlap, suggesting that class attribution can approximate expert reasoning when class boundaries are distinct. Perceptual metrics (LPIPS and DreamSim) were informative but sensitive to feature extractor and task configuration. MD consistently failed to capture semantic structure, likely due to violations of class-conditional Gaussian assumptions in high-dimensional feature spaces with substantial intra-class variability.^45^^,^^46^^,^^47^ No metric fully substituted expert assessment, as increasing morphological complexity reduced both metric alignment and inter-expert agreement, reflecting intrinsic diagnostic ambiguity rather than metric limitations alone. Beyond methodological interpretability, while CVs and AAs are not direct clinical decision-making tools, they may support model evaluation, auditing, and trust building by making learned representations inspectable before clinical deployment. Experts could use them to assess whether model-derived concepts reflect plausible histomorphology or instead indicate artifacts, staining variation, or dataset-specific shortcuts. Atlas organization may also inform dataset curation and class-taxonomy design by revealing morphologically incoherent, overly fine-grained, heterogeneous, or inconsistently labeled classes. In human-in-the-loop validation, such visualizations may support assessment of biological plausibility, task relevance, and consistency with known histomorphological correlates, while also highlighting candidate morphological patterns for targeted validation. They should, therefore, be regarded as complementary auditing tools rather than evidence of clinical correctness.

### Limitations of the study

Limitations include the reliance on UNI as a single pathology foundation model, a frozen-backbone training regime, and the sensitivity of feature visualizations to architecture, layer selection, optimization strategy, and dimensionality reduction.^29^^,^^30^^,^^36^ All experiments were performed with UNI and a task-specific linear head; therefore, the observed atlas organization, layer-wise progression, and relationship between visual separability and expert ambiguity should be interpreted as properties of this specific model and training configuration rather than as general characteristics of all transformer-based pathology models. End-to-end fine-tuning could reshape internal representations and potentially increase task-specific separability, but might also make it harder to distinguish pretrained morphological structure from task-induced features. Conversely, CNN-based models or alternative pathology foundation models may organize morphology differently because of differences in architectural inductive bias, receptive field structure, and token- or feature-level aggregation.^36^^,^^48^^,^^49^ With respect to dimensionality reduction, t-SNE emphasizes local neighborhood structure rather than global distance preservation; atlas proximity, cluster boundaries, and apparent separability should be interpreted as exploratory features of the two-dimensional embedding rather than measurements of the original activation space.^50^^,^^51^ Like other low-dimensional embedding methods, t-SNE is sensitive to hyperparameters and initialization, and different embedding choices may alter atlas topology; moreover, any two-dimensional projection necessarily discards information from the original high-dimensional representation space.^52^ Generated images are further constrained by the training distribution, optimization procedure, and analyzed image tiles and should be interpreted as exploratory summaries of learned morphological patterns rather than as causal explanations of decision mechanisms. As the study focused on tile-level morphology, sex- and gender-associated differences and the influence of other patient-level variables, such as disease stage, were not assessed; their relationship with learned morphological representations warrants future investigation. Moreover, latent features remain abstract and interact non-linearly, risking overinterpretation.^53^ Quantitative evaluation is further constrained by the absence of pathology-specific perceptual metrics; LPIPS and DreamSim were developed for natural images and emphasize global structure over subtle cellular morphology.^54^^,^^55^ Domain-specific fine-tuning grounded in expert similarity judgments could improve metric validity.

Future work should extend this framework to narrowly defined differentiation tasks or molecular subtypes to enable validation against established genotype-phenotype correlations and identify residual, potentially underrecognized features.^15^^,^^22^ Scaling from patches to WSIs would facilitate analysis of spatial context and multiscale organization, contingent on computational feasibility. Robustness should be assessed across image initializations, optimization settings, and embedding methods, while systematic comparisons across layers, backbone architectures, classifier heads, and training regimes may clarify how architectural and training choices shape morphological representation hierarchies. Finally, development of pathology-specific perceptual metrics remains essential to better align quantitative similarity measures with expert judgment.

In conclusion, concept-level feature visualization provides a structured characterization of how the UNI-based pathology model evaluated here encodes morphological information. By revealing overlap, continuity, and task-dependent separability within representation spaces, it reflects intrinsic morphological complexity and expert agreement across label granularities. This framework advances interpretability beyond single-prediction explanations by enabling systematic analysis of internal representation structure and, through its modular design, facilitating the exchange of model backbones and classifier heads for comparative analyses across foundation-model architectures, training regimes, and downstream tasks. It may thereby support transparent model evaluation, help identify limits of class separability, and facilitate informed human-AI interaction in computational pathology.

## Resource availability

### Lead contact

Further information and requests should be addressed to the lead contact, Jakob Nikolas Kather (kather.jn@tu-dresden.de).

### Materials availability

This study did not generate new unique reagents or biological materials.

### Data and code availability

All data used in this study are publicly available. The NCT-CRC-HE-100K and CRC-VAL-HE-7K datasets^41^ are available at https://zenodo.org/records/1214456. The TCGA-based cancer classification datasets derived from The Cancer Genome Atlas (TCGA)^42^^,^^43^ are available at https://zenodo.org/records/5889558. For the exploratory normal adjacent tissue versus tumor classification experiment, TCGA whole-slide images and associated data were accessed through the Genomic Data Commons (GDC) Data Portal at https://portal.gdc.cancer.gov/. The source code for model training, class visualization, activation atlas generation, and interactive atlas exploration is publicly available at https://github.com/KatherLab/PathoActivationAtlas. Any additional information required to reanalyze the data reported in this paper is available from the lead contact upon request.

## Acknowledgments

The authors are grateful to the Center for Information Services and High Performance Computing (10.13039/501100019676Zentrum für Informationsdienste und Hochleistungsrechnen, Technische Universität Dresden [ZIH]) at TU Dresden for providing its facilities for high-throughput calculations. Aspects of this work are part of the medical doctoral thesis of K.A. J.N.K. is supported by the German Cancer Aid DKH (DECADE, 70115166), the German Federal Ministry of Research, Technology and Space BMFTR (PEARL, 01KD2104C; CAMINO, 01EO2101; TRANSFORM LIVER, 031L0312A; and TANGERINE, 01KT2302, through ERA-NET Transcan; Come2Data, 16DKZ2044A; DEEP-HCC, 031L0315A; DECIPHER-M, 01KD2420A; NextBIG, 01ZU2402A; and PROSURV, 01KD2509C), the 10.13039/501100001659German Research Foundation (DFG, 10.13039/501100001659Deutsche Forschungsgemeinschaft) as part of Germany’s Excellence Strategy – EXC 2050/2 – Project ID 390696704 – Cluster of Excellence “Centre for Tactile Internet with Human-in-the-Loop” (CeTI) of 10.13039/501100002957Technische Universität Dresden, as well as through 10.13039/501100001659DFG-funded collaborative research projects (TRR 412/1, 535081457; SFB 1709/1 2025, 533056198), the German Academic Exchange Service DAAD (SECAI, 57616814), the German Federal Joint Committee G-BA (TransplantKI, 01VSF21048), the 10.13039/501100000780European Union’s Horizon Europe research and innovation program (ODELIA, 101057091; GENIAL, 101096312), the European Research Council ERC (NADIR, 101114631), the 10.13039/100001006Breast Cancer Research Foundation (BELLADONNA, BCRF-25–225), and the 10.13039/501100000272National Institute for Health and Care Research NIHR (10.13039/501100018955Leeds Biomedical Research Centre, NIHR203331). The views expressed are those of the authors and not necessarily those of the NHS, the NIHR, or the Department of Health and Social Care. This work was funded by the European Union. Views and opinions expressed are, however, those of the authors only and do not necessarily reflect those of the European Union. Neither the European Union nor the granting authority can be held responsible for them. N.G.R. is supported by the Clinician Scientist Programme of the Faculty of Medicine, University of Augsburg, Germany. S.F. is supported by the 10.13039/501100002347Federal Ministry of Research, Technology and Space (DECIPHER-M, 01KD2420E), the 10.13039/501100005972German Cancer Aid (DECADE, 70115166 and TargHet, 70115995).

## Author contributions

M.G., F.W., C.G., N.G.R., S.F., and J.N.K. conceptualized and designed the study. M.G., F.W., and C.G. developed the methodological framework for concept-level feature visualization. M.G. and F.W. curated and preprocessed the source datasets. F.W. implemented the deep learning training and inference pipeline. C.G. and F.W. developed the code for class visualizations and activation atlases. C.G. implemented quantitative evaluation metrics. M.G., C.G., and F.W. developed the software for data analysis, visualization, and interactive exploration tools. M.G., C.G., and F.W. planned and conducted the computational experiments. M.G. performed the analysis and interpretation of model outputs and visualization of results. N.G.R., S.F., S.S., K.A., and B.M. conducted pathological examination of samples and interpreted generated visualizations. M.G., N.G.R., S.F., and J.N.K. contributed to the interpretation of results in a clinical and methodological context. M.G. drafted the initial manuscript. M.G. prepared figures and supplementary materials. All authors revised the manuscript critically for important intellectual content, jointly interpreted the data, and approved the final version of the manuscript for submission.

## Declaration of interests

J.N.K. declares ongoing consulting services for AstraZeneca and Bioptimus. Furthermore, he holds shares in StratifAI, Synagen, and Spira Labs and has received institutional research grants from GSK and AstraZeneca as well as honoraria from AstraZeneca, Bayer, Daiichi Sankyo, Eisai, Janssen, Merck, MSD, BMS, Roche, Pfizer, and Fresenius. M.G. received honoraria for lectures sponsored by Sartorius AG, Germany, and AstraZeneca, UK. S.F. received honoraria from AstraZeneca, BMS, and MSD. N.G.R. received travel support from Bruker Spatial Biology.

## Declaration of generative AI and AI-assisted technologies in the writing process

After preparing the initial manuscript, the authors used ChatGPT (OpenAI, GPT-5.5) and DeepL Write (DeepL SE) to edit selected sections to improve readability. After using these tools, the authors carefully reviewed and edited the text as needed and take full responsibility for the content of the publication.

## STAR★Methods

### Key resources table

**Table:** 

REAGENT or RESOURCE	SOURCE	IDENTIFIER
**Software and algorithms**		

Study Repository: PathoActivationAtlas	GitHub	https://github.com/KatherLab/PathoActivationAtlas; Commit: https://github.com/KatherLab/PathoActivationAtlas/tree/fb0d787af210a8afbedeec98af6f71d5817cbb62
UNI	Hugging Face	https://huggingface.co/MahmoodLab/UNI
H-optimus-0	Hugging Face	https://huggingface.co/bioptimus/H-optimus-0
Prov-GigaPath	Hugging Face	https://huggingface.co/prov-gigapath/prov-gigapath
Python 3.12.7/3.14.2 (statistics)	Python	https://www.python.org, RRID:SCR_008394
PyTorch 2.6.0	Python	https://pytorch.org, RRID:SCR_018536
TorchVision 0.21.0	Python	https://pytorch.org/vision
NumPy 1.26.4/2.4.0 (statistics)	Python	https://numpy.org, RRID:SCR_008633
Pandas 2.2.2/2.3.3 (statistics)	Python	https://pandas.pydata.org, RRID:SCR_018214
SciPy 1.13.0	Python	https://scipy.org, RRID:SCR_008058
Scikit-learn 1.4.2/1.8.0 (statistics)	Python	https://scikit-learn.org, RRID:SCR_002577
Matplotlib 3.8.3/3.10.8 (statistics)	Python	https://matplotlib.org, RRID:SCR_008624
Seaborn 0.13.2	Python	https://seaborn.pydata.org, RRID:SCR_018132
OpenCV Python 4.9.0.80	Python	https://opencv.org, RRID:SCR_015526
Pillow 10.3.0/12.1.0 (statistics)	Python	https://python-pillow.org, RRID:SCR_023337
Timm 1.0.11	GitHub	https://github.com/huggingface/pytorch-image-models
Transformers 4.46.3	Hugging Face	https://huggingface.co/docs/transformers, RRID:SCR_027381
Torch Lucent 0.1.8	GitHub	https://github.com/greentfrapp/lucent
DreamSim 0.2.1	GitHub	https://github.com/ssundaram21/dreamsim
LPIPS 0.1.4	Python	https://github.com/richzhang/PerceptualSimilarity
Clean-FID 0.1.35	Python	https://github.com/GaParmar/clean-fid
Captum 0.5.0	Python	https://captum.ai
UMAP-learn 0.5.5	Python	https://umap-learn.readthedocs.io, RRID:SCR_018217
PyTorch Lightning 2.2.4	Python	https://lightning.ai, RRID:SCR_027468
statsmodels 0.14.6	Python	https://www.statsmodels.org, RRID:SCR_016074
krippendorff 0.8.2	Python	https://pypi.org/project/krippendorff/
Plotly 6.5.1	Python	https://plotly.com/python/, RRID:SCR_013991

**Other**		

NCT-CRC-HE-100K Dataset	Zenodo	https://zenodo.org/records/1214456
CRC-VAL-HE-7K Dataset	Zenodo	https://zenodo.org/records/1214456
TCGA-UT Dataset	Zenodo	https://zenodo.org/records/5889558
TCGA Dataset for Revision Experiments	Genomic Data Commons Data Portal	https://portal.gdc.cancer.gov/

### Experimental model and study participant details

#### Histopathology datasets

In this study we analyzed model activations on two morphologically diverse H&E datasets (Figure 2). The NCT-CRC-HE-100K dataset (Figure 2A), hereafter NCT dataset, has been widely adopted in prior computational pathology studies^41^ and comprises 100,000 non-overlapping 224 × 224 pixels (px) patches (0.5 μm/px) from 86 colorectal cancer slides, manually annotated into nine tissue classes including tumor and non-tumor regions (Table S1; Figure 2A). Images were color-normalized using Macenko’s method.^56^ The second dataset, derived from The Cancer Genome Atlas^42^^,^^43^ (Figure 2B, dataset publicly available under https://www.cancer.gov/tcga), hereafter referred to as TCGA, contains 1,608,060 patches randomly sampled from annotated tumor regions across 32 cancer types, of which eleven were selected to represent clinically relevant and morphologically diverse entities (Table S1; Figure 2B). Patches were extracted at 0.5 μm/px with a size of 256 × 256 px without color normalization. Public access links for datasets and code are provided in the data and code availability section. For the exploratory normal adjacent tissue versus tumor dataset, a board-certified pathologist (SF) selected TCGA slides from five carcinoma subtypes (colon adenocarcinoma (COAD), breast invasive carcinoma (BRCA), lung adenocarcinoma (LUAD), prostate adenocarcinoma (PRAD), liver hepatocellular carcinoma (LIHC)) that contained representative carcinoma and matched normal adjacent tissue regions. Patches were color-normalized using Macenko’s method to reduce slide-level stain variability and support comparison between normal adjacent and tumor regions. This study was performed in accordance with the Declaration of Helsinki and represents a retrospective analysis of scanned images of anonymized tissue samples from various cohorts of cancer patients. The analyzed data were obtained from publicly available repositories in anonymized form. The overall analysis was approved by the Ethics Board of the Medical Faculty of TUD Dresden University of Technology under the ID BO-EK-444102022.

### Method details

#### Image processing and model training

Feature visualization involves generating images that activate specific features within a defined layer in a neural network; CV, as a subset of this, focuses on maximizing the activation in the final layer relevant to predicting a particular tissue or cancer class. Generated feature visualizations depend on the network architecture, learned parameters, selected layer, and optimization hyperparameters, and, for AAs, on the dataset from which target activations are derived. They should therefore be interpreted as model- and dataset-specific conceptual representations rather than faithful reconstructions of internal decision mechanisms. For training and activation capture, we employed the ViT-based foundation model UNI as backbone^25^^,^^39^ with a subsequent linear classification layer that outputs class logits. The final linear layer was trained using a cross-entropy loss function, with frozen backbone weights. For consistency, the same model architecture was applied across all experiments, varying only the training dataset. We used a frozen-backbone setting to reflect common computational pathology workflows, where foundation models serve as general-purpose feature extractors with lightweight task-specific classifiers. This enabled interrogation of the pretrained UNI representation space while limiting confounding from end-to-end fine-tuning. Accordingly, the visualizations are specific to this UNI-based feature-extraction setting rather than universal properties of transformer architectures or training regimes. The modular visualization pipeline further allows exchange of model backbones and classifier heads, supporting broader adoption and systematic testing across foundation-model architectures.

In detail, TCGA patches were randomly cropped to 224 × 224 px to match the input dimensions of the ViT architecture. A unified preprocessing pipeline was applied, including random flipping, rotation, and color jitter (brightness, contrast, and saturation), followed by normalization using ImageNet statistics (mean [0.485, 0.456, 0.406], standard deviation [0.229, 0.224, 0.225]). The NCT dataset was partitioned into 5 folds for cross validation, using 4 folds for training and one for validation in each iteration. The CRC-VAL-HE-7K dataset^41^ was reserved for external testing. TCGA patches were grouped into 6 folds using StratifiedGroupKFold^57^ to prevent patch-level data leakage; 1 fold was held out for testing and the remaining five were used for cross validation. Models were trained using the AdamW optimizer^58^ with a learning rate of 0.001 for up to 50 epochs, with early stopping after 20 epochs without improvement in validation loss. Although 5-fold cross validation was used to estimate performance, feature and class visualizations were generated from a single model to limit computational cost, specifically the checkpoint with the lowest validation loss from the first training fold.

We adapted CVs and AAs for histopathology to expose model-relevant morphological concepts rather than provide mechanistic explanations of model internals.^29^^,^^36^^,^^37^ Both methods optimize an input image to induce predefined target activations (Figure 1), either by maximizing a class logit (CV) or by matching previously captured and aggregated activations (AA). The selected network layer determines feature complexity, with earlier layers encoding simple structures and deeper layers integrating more complex patterns.^29^^,^^37^ The target activation vector *f* may correspond to the full layer output or a subset, such as a specific token (e.g., the class token (cls)) or class logit. In contrast to model training, parameters remain fixed and the input image is iteratively optimized. Images were initialized randomly and parameterized in Fourier space to reduce high-frequency artifacts and improve convergence, followed by inverse Fourier transform to obtain spatial domain visualizations.

#### Class visualizations

Class Visualization (CV) generates class-specific feature representations by optimizing an input image to maximize a target class logit^36^ (Figure 1B). For each class *c* in the NCT and TCGA datasets, we optimized an image *img* such that *img*∗ = *argmax*_*img*_*cl*(*p*,*c*), where *cl*(*p*,*c*) denotes the pre-softmax logit for class *c* under model parameters *p*.^27^ Optimization of the raw class logits produced the most coherent and recognizable images and was performed via backpropagation for 8192 iterations, after which convergence was consistently observed.

#### Activation atlases

Feature inversion was used to generate Activation Atlases (AAs) by reconstructing randomly initialized input images from internal activations, enabling analysis of concept organization within the model’s representations rather than individual predictions.^36^ Activations corresponding to a predefined target feature vector *f*∗ at layer *l* of the trained transformer with parameters *p* were first extracted for all training patches. These high-dimensional activations were embedded into two dimensions using t-distributed stochastic neighbor embedding (t-SNE)^50^ (Figures 1D and 1E) and partitioned the embedding into grids of 10 × 10 for NCT and 20 × 20 for TCGA, respectively.

t-SNE was selected to visualize local activation neighborhoods and gradual feature transitions, rather than exact global distances; atlas proximity and separability should therefore be interpreted as exploratory summaries, not quantitative measures of global representational distance.^51^ Because distances in high-dimensional spaces are difficult to interpret and any two-dimensional embedding distorts the original feature space, PCA was considered less suitable because it captures only linear variation, whereas high-dimensional activations may contain nonlinear structure. UMAP was considered as an alternative nonlinear method, but in preliminary inspection yielded compact, separated clusters with larger empty regions, limiting visualization of gradual transitions in densely populated activation atlases. Based on the resulting t-SNE grid, original activations within each cell were averaged component-wise to aggregated target vectors. For each cell, an image *img*∗ was optimized to match its target activation *f*∗ by minimizing ${img}^{*}={\arg\;\min}_{img}\;L\left(f_{l}\left(img;p\right),f^{*}\right)$^37^^,^^53^, where *f*_*l*_(*img*;*p*) denotes the activation at layer *l*. We used the Euclidean (L2) loss $L\left(f_{l}\left(img;p\right),f^{*}\right)={\left\|f_{l}\left(img;p\right)-f^{*})\right\|}_{2}^{2}$ and performed optimization via backpropagation for 8192 iterations until convergence. Optimized images were arranged according to their grid position to form the atlas.

For each cell, we additionally recorded the class distribution of underlying ground-truth (GT) patches and computed class attribution scores.^36^ For a given class *c*, the attribution was defined as the mean dot product

$$\frac{\Sigma_{i}\;<f_{i}^{*},\nabla_{f_{i}^{*}}\;cl\left(p,c\right)>}{n}$$

where <·,·> denotes the dot product, $f_{i}^{*}$ represents one of the cell’s *n* individual target activations prior to averaging, and $\nabla_{f_{i}^{*}}\;cl\left(p,c\right)$ is the gradient of the class logit with respect to that activation. Thus, each cell’s attribution score corresponds to the mean contribution of its unaggregated activations to the specified class logit. AAs and associated metadata can be explored using the interactive visualization tool provided with this study, as described in the data and code availability section. Feature visualization was performed by extracting activations from selected layers of trained models using 224 × 224 px input patches without augmentation. Unless otherwise specified, activations were obtained from an intermediate layer (layer 14 of 24) of the UNI model for AA generation, as earlier layers primarily encoded low-level features, while deeper layers produced increasingly specialized and fragmented representations; for one model, all 24 layers were analyzed for comparison. In total, four models were trained: one on the NCT dataset including all nine tissue classes, and three on TCGA (Table S1) using clinically defined subsets. These comprised TCGA-5, including sarcoma (SARC), diffuse large B cell lymphoma (DLBCL), colorectal adenocarcinoma (COAD/READ), and melanoma (SKCM); TCGA-8, including lung squamous cell carcinoma (LUSC), lung adenocarcinoma (LUAD), breast carcinoma (BRCA), prostate adenocarcinoma (PRAD), colorectal adenocarcinoma (COAD/READ), kidney renal clear cell carcinoma (KIRC), and kidney renal papillary cell carcinoma (KIRP); and TCGA-11, combining all classes from TCGA-5 and TCGA-8. CVs were generated for the NCT (9 classes) and TCGA-11 (11 classes) models. As an exploratory extension to assess organ-specific non-neoplastic tissue context, CVs and AAs were generated for the normal adjacent tissue versus tumor classification experiment using the same workflow as described above.

#### Pathologist annotation workflow

Four pathologists, including one board-certified pathologist, independently annotated real H&E-stained patch images, class visualizations, and activation atlas cell images from the NCT and TCGA tasks. Images were presented in randomized order, and annotators were blinded to reference labels and metric-derived assignments. For each image, annotators selected one task-specific class label or marked the image as uncertain. Reference labels for generated images were defined as the optimized target class for class visualizations and, for activation atlas cells, as the majority ground-truth class of the real patches contributing to the aggregated target activation. Uncertain annotations were either excluded or treated as a separate category, depending on the analysis.

#### Quantitative surrogate metrics

The generated images visualized model-relevant features, enabling pathologists to assess correspondence with established morphological patterns and to identify potentially unrecognized diagnostic features. Because concept-level visualizations lacked a single objective GT, we evaluated alignment with inter-observer agreement rather than predictive accuracy or causal faithfulness. We assessed agreement and classification performance on real and generated samples. A generated image’s GT label was defined as the class it was optimized to visualize (CV) or as the majority GT class of the real samples from which the target activation was aggregated (AA). We then tested whether class attribution and quantitative metrics could serve as surrogates for pathologists’ assessments by measuring their agreement with pathologist classifications and GT labels.

Conventional metrics such as mean squared error (MSE) and structural similarity index measure (SSIM) do not capture the context-dependent complexity of human visual perception.^54^^,^^55^^,^^59^ Perceptual similarity is difficult to formalize because human judgments do not strictly satisfy mathematical properties of a distance metric.^55^ We therefore used DL-based feature embeddings (e.g., UNI^25^ for H&E images) to approximate perceptual similarity, particularly for complex image generation.^54^ Except for attributions, all applied metrics were distance measures computed on features extracted from real and generated images, including Mahalanobis distance, learned perceptual image patch similarity (LPIPS), and DreamSim.

Mahalanobis Distance (MD) measures a distance between a point and a distribution of such points, assuming a class-conditional Gaussian distribution.^46^^,^^47^^,^^60^^,^^61^ The MD of a point $P\in R^{N}$ from a distribution with mean $\mu \in R^{N}$ and covariance matrix $\Sigma \in R^{N\times N}$ is defined as $\sqrt{{\left(P-\mu \right)}^{T}\Sigma^{-1}\left(P-\mu \right)}$. Unlike Euclidean distance (ED), MD accounts for feature correlations, which improves performance in high-dimensional spaces.^46^^,^^47^ For numerical stability, we estimated covariance matrices using Ledoit-Wolf shrinkage^45^ and computed their pseudo-inverse. We computed squared MDs between feature vectors of generated images and the class-specific distribution of sampled real-image features. Features were extracted using UNI, H-optimus-0,^62^ and Prov-GigaPath.^63^ Lower MD indicated greater class consistency from the respective feature space. Due to memory constraints, we used only the final feature representations.

Learned Perceptual Image Patch Similarity (LPIPS) measures a distance between two images *img*_1_, *img*_2_ based on features extracted from multiple layers of a pre-trained CNN.^54^^,^^55^ A lower LPIPS value corresponds to higher image similarity. The LPIPS value is calculated as

$$LPIPS\left({img}_{1},{img}_{2}\right)=\sum_{l}\frac{1}{H_{l}W_{l}}\sum_{h,w}{\left\|w_{l}⊙\left({\hat{f}}_{1,l}^{h,w}-{\hat{f}}_{2,l}^{h,w}\right)\right\|}_{2}^{2}$$

with ${\hat{f}}_{1,l}^{h,w}$, ${\hat{f}}_{2,l}^{h,w}\in R^{H_{l}\times W_{l}\times C_{l}}$ being features from layer *l* at spatial position (*h*, *w*) that have been unit-normalized across all *C*_*l*_ channels. $w_{l}\in R^{C_{l}}$ denotes channel-wise weights trained for image similarity. For transformer-based architectures, we replaced spatial dimensions (*H*_*l*_*W*_*l*_) with the token dimension and indexed positions by token. For linear layers, we set *H*_*l*_*W*_*l*_ = 1. LPIPS distances were computed between generated images and real images for each GT class using AlexNet,^64^ UNI, H-Optimus-0, and Prov-GigaPath. For AlexNet, we adopted the layer selection and pretrained perceptual weights proposed by Zhang et al.^55^ Although AlexNet was not trained on pathology images, the three pathology-specific networks were included to mitigate this limitation. For the transformer-based models, we did not train the layer-wise weights *w*_*l*_ and fixed them to 1 (i.e., identity weighting).

DreamSim^54^ is a learned perceptual metric that computes image distance from concatenated embeddings of DINO,^65^ CLIP^66^ and OpenCLIP.^67^ The ensemble was fine-tuned on mid-level variations (e.g., layout, pose, semantic content) to better align with human perception than metrics such as LPIPS. Given two images *img*_1_ and *img*_2_ with feature vectors ${\hat{f}}_{1},{\hat{f}}_{2}\in R^{C}$, the distance is defined as

$$DreamSim\left({img}_{1},{img}_{2}\right)=1-\frac{<{\hat{f}}_{1},{\hat{f}}_{2}>}{{\left\|{\hat{f}}_{1}\right\|}_{2}{\left\|{\hat{f}}_{2}\right\|}_{2}}$$

i.e., as the cosine distance between the two feature vectors. A lower DreamSim value indicates higher image similarity. As fine-tuning DreamSim on pathology images was beyond the scope of this study, we used the pretrained model to compute distances between generated images and real images for each GT class.

### Quantification and statistical analysis

#### Model performance

To verify that the trained classifiers provided a suitable basis for feature visualization, model performance was evaluated on held-out test data. We reported area under the receiver operating characteristic curve (AUROC), F1 score, and accuracy for the NCT tissue classification task and the TCGA cancer classification tasks. These metrics were used only to confirm adequate classifier performance before generating class visualizations and activation atlases, not as the primary endpoint of the interpretability analysis.

#### Annotation performance and agreement

Statistical analyses quantified whether expert annotations of real and generated images reflected shared, human-recognizable morphological concepts rather than diagnostic accuracy or explanation correctness. Inter-annotator agreement was assessed using Fleiss’ κ for overall agreement, Cohen’s κ for pairwise annotator and annotator–metric agreement, and Krippendorff’s α for nominal agreement allowing missing labels. Agreement was interpreted as consistency of morphological interpretation, not model validation. For activation atlas images, agreement was computed between annotator pairs and between annotators and atlas-induced labels obtained using nearest-neighbor and distance-based feature aggregation. Uncertainty for Cohen’s κ and percent agreement was estimated by bootstrap resampling of paired image-level labels with 300 iterations and a fixed seed; Krippendorff’s α was computed on the full annotation matrix. Accuracy, F1 score, sensitivity, specificity, and confusion matrices were calculated descriptively against reference labels to assess class-discriminative information. Inference was performed at the image level and is therefore conditional on the four participating pathologists.

#### Scan-CV permutation testing

To objectively compare the recognizability of real scan images and CVs summarized in Figure 5, we tested scan–CV differences for all performance and agreement metrics using item-level resampling. Because only four annotators were available and κ- and α-based metrics lack closed-form standard errors, inference was performed at the image level rather than the rater level. For each dataset (NCT, TCGA-11, and TCGA-11-adeno), uncertainty setting, and metric, we calculated the effect size as Δ = metric(scans) − metric(CVs), yielding 42 comparisons. Two-sided *p* values were obtained by stratified permutation testing with 10,000 permutations, permuting scan/CV membership within each reference class while preserving class sizes. Confidence intervals (95%) were estimated by stratified bootstrap resampling with 10,000 iterations, resampling scans and CVs independently within each reference class. *p* values were Holm-corrected across all 42 comparisons, with significance defined as Holm-adjusted *p* < 0.05. Because no permutation reached the observed effect in any comparison, *p* values are reported as upper bounds at the permutation-resolution limit (raw *p* < 0.0001; Holm-adjusted *p* < 0.005).

## Supplemental information

Document S1. Figures S1–S18 and Tables S1 and S2

Document S2. Article plus supplemental information
