# Supplementary material for: Class visualizations and activation atlases for computational pathology
Source: Cell Rep Med. 2026 Sep 18;7(10):103054. doi: 10.1016/j.xcrm.2026.103054 (PMC13710382; doi:10.1016/j.xcrm.2026.103054)
Supplement: Supplementary file 1 [file mmc1.pdf]

Cell Reports Medicine, Volume *7*

Supplemental information

Class visualizations and activation atlases

for computational pathology

Marco Gustav, Fabian Wolf, Christina Glasner, Nic G. Reitsam, Stefan Schulz, Kira Aschenbroich, Bruno Märkl, Sebastian Foersch, and Jakob Nikolas Kather

Supplementary Figures

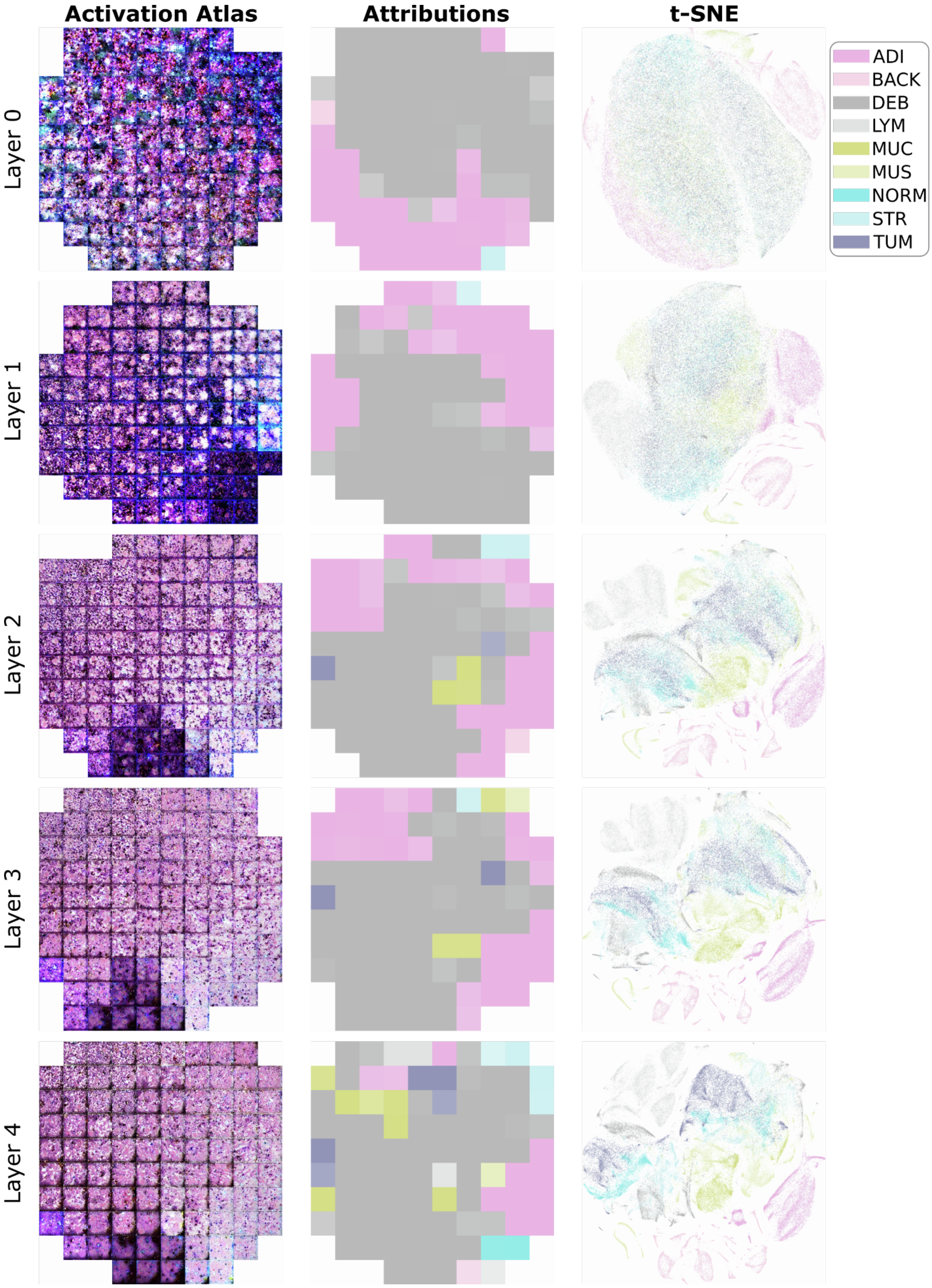

Figure S1 | Activation atlases, class attributions, and embedding projections for layers 0–4 in the National Center for Tumor Diseases (NCT) dataset. Related to Figure 6.
Activation atlases, class attribution maps, and two-dimensional t-distributed stochastic neighbor embedding (t-SNE) projections derived from layers 0–4 (of a total of 24 layers) of the transformer-based UNI model trained

on the NCT dataset. Each row corresponds to one network layer. The first column shows activation atlases generated by feature inversion, summarizing aggregated activation patterns across the embedding space. The second column shows tissue-type attribution maps indicating the dominant tissue class associated with each activation atlas cell. The third column shows t-SNE projections of patch-level feature embeddings extracted from the same layer, colored by tissue class. Class color codes are shown on the right, assigning one of the nine tissue classes: adipose tissue (ADI), background (BACK), debris (DEB), lymphocytes (LYM), mucus (MUC), smooth muscle (MUS), normal colon mucosa (NORM), cancer-associated stroma (STR), and colorectal adenocarcinoma epithelium (TUM).

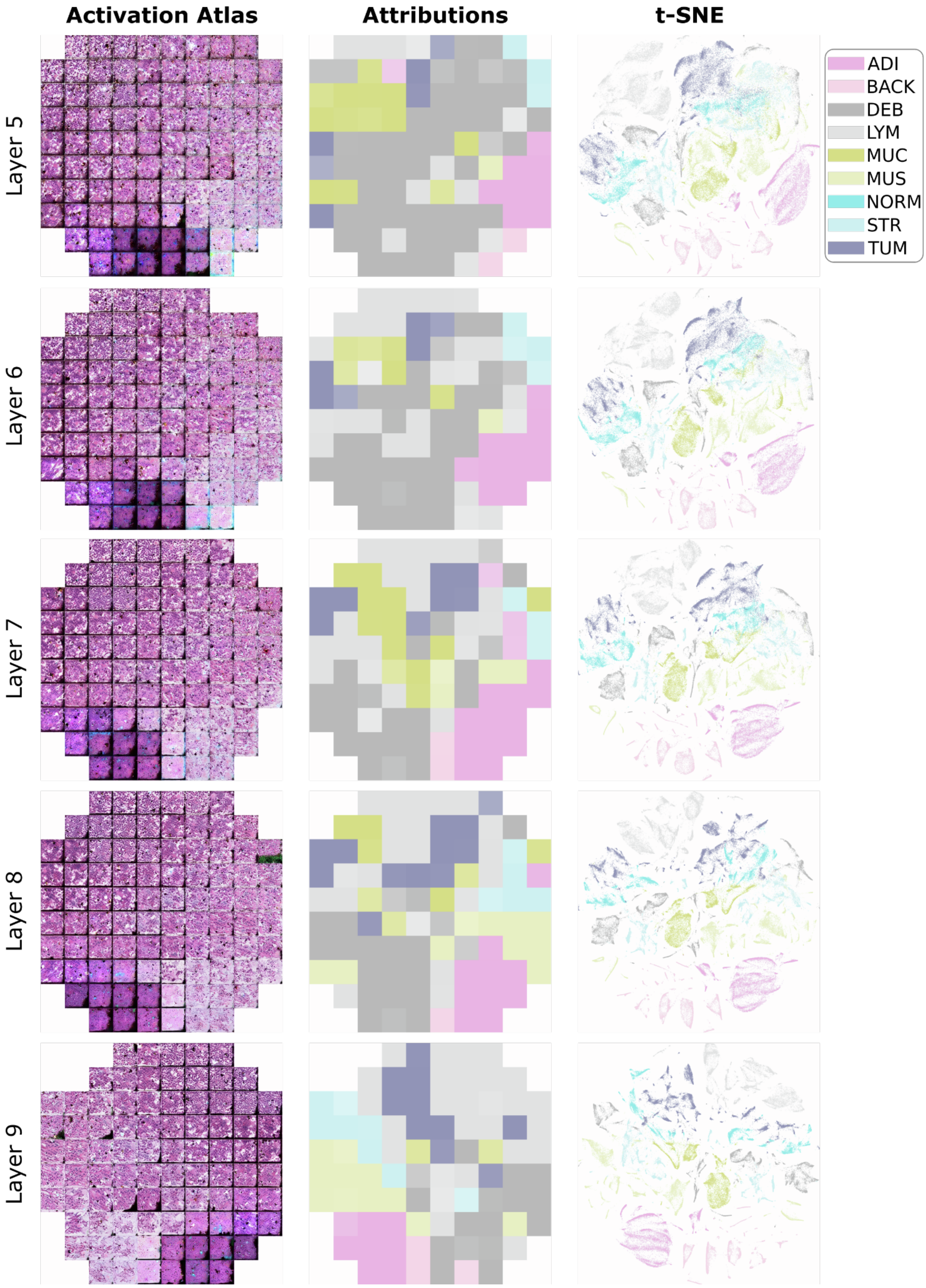

Figure S2 | Activation atlases, class attributions, and embedding projections for layers 5–9 in the National Center for Tumor Diseases (NCT) dataset. Related to Figure 6.
Activation atlases, class attribution maps, and two-dimensional t-distributed stochastic neighbor embedding (t-SNE) projections derived from layers 5–9 (of a total of 24 layers) of the transformer-based UNI model trained on the NCT dataset. Each row corresponds to one network layer. The first column shows activation atlases generated by feature inversion, summarizing aggregated activation patterns across the embedding space. The second column shows tissue-type attribution maps indicating the dominant tissue class associated with each activation

atlas cell. The third column shows t-SNE projections of patch-level feature embeddings extracted from the same layer, colored by tissue class. Class color codes are shown on the right, assigning one of the nine tissue classes: adipose tissue (ADI), background (BACK), debris (DEB), lymphocytes (LYM), mucus (MUC), smooth muscle (MUS), normal colon mucosa (NORM), cancer-associated stroma (STR), and colorectal adenocarcinoma epithelium (TUM).

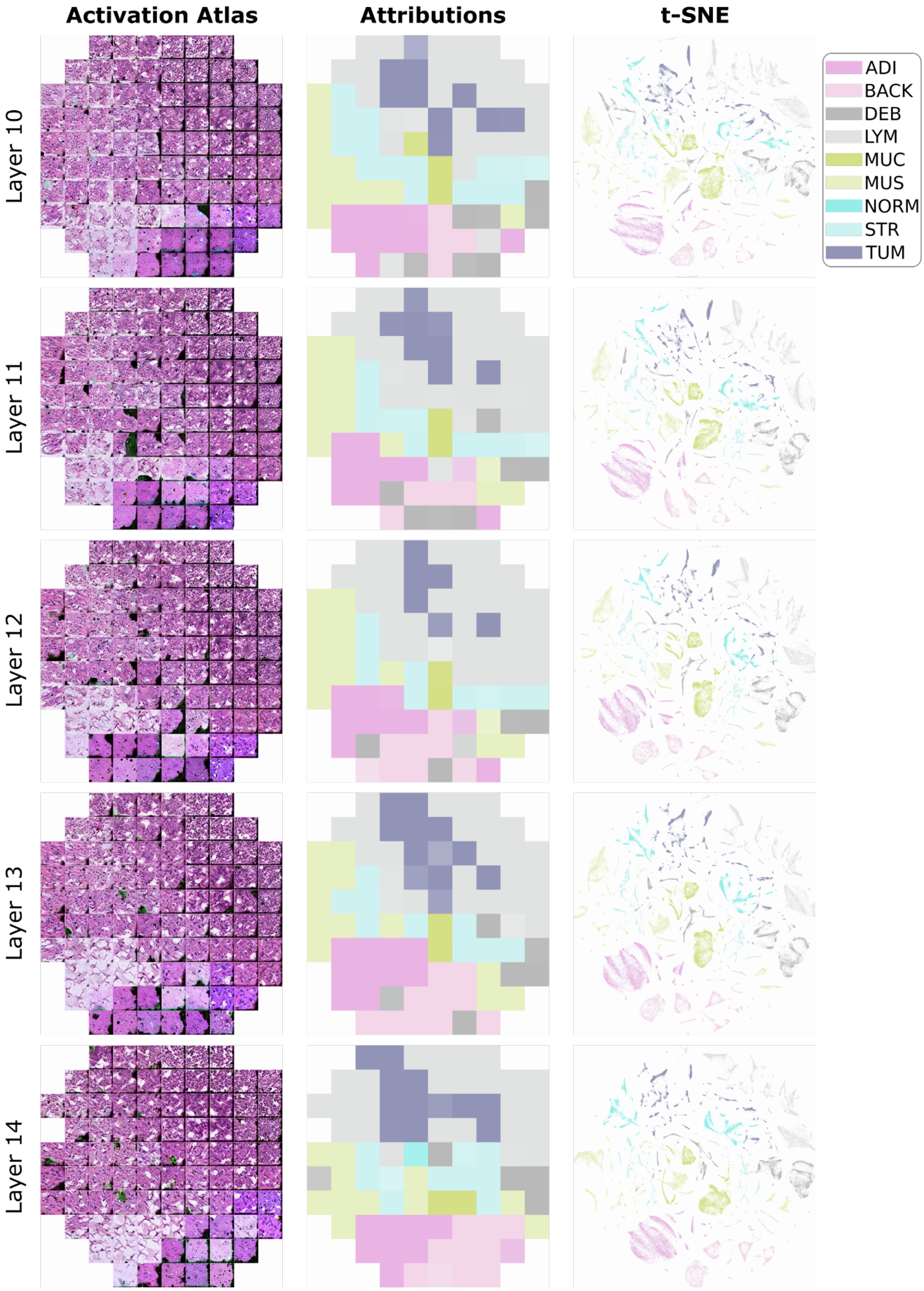

Figure S3 | Activation atlases, class attributions, and embedding projections for layers 10–14 in the National Center for Tumor Diseases (NCT) dataset. Related to Figure 6.

Activation atlases, class attribution maps, and two-dimensional t-distributed stochastic neighbor embedding (t-SNE) projections derived from layers 10–14 (of a total of 24 layers) of the transformer-based UNI model trained on the NCT dataset. Each row corresponds to one network layer. The first column shows activation atlases generated by feature inversion, summarizing aggregated activation patterns across the embedding space. The second column shows tissue-type attribution maps indicating the dominant tissue class associated with each activation

atlas cell. The third column shows t-SNE projections of patch-level feature embeddings extracted from the same layer, colored by tissue class. Class color codes are shown on the right, assigning one of the nine tissue classes: adipose tissue (ADI), background (BACK), debris (DEB), lymphocytes (LYM), mucus (MUC), smooth muscle (MUS), normal colon mucosa (NORM), cancer-associated stroma (STR), and colorectal adenocarcinoma epithelium (TUM).

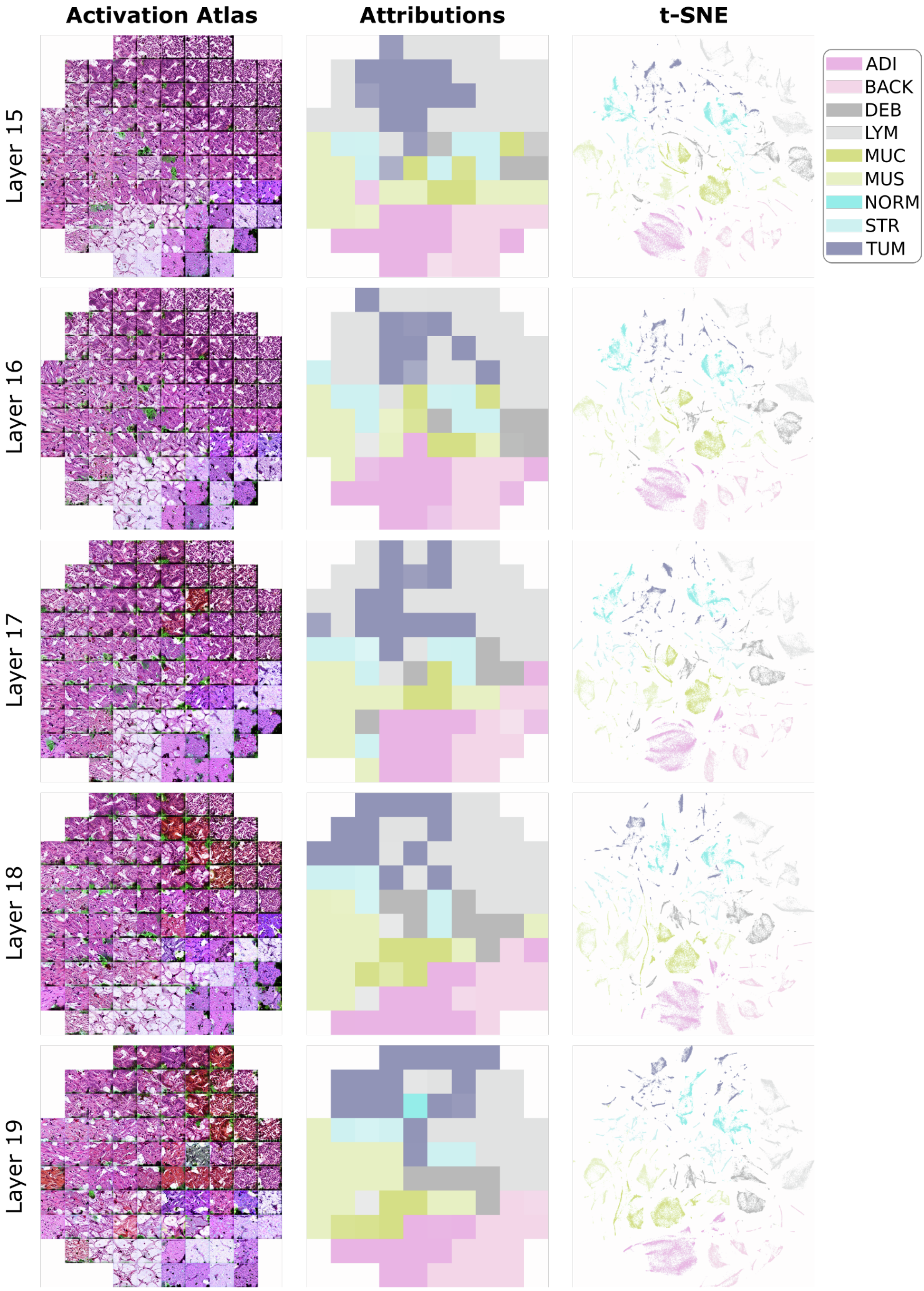

Figure S4 | Activation atlases, class attributions, and embedding projections for layers 15–19 in the National Center for Tumor Diseases (NCT) dataset. Related to Figure 6.
Activation atlases, class attribution maps, and two-dimensional t-distributed stochastic neighbor embedding (t-SNE) projections derived from layers 15–19 (of a total of 24 layers) of the transformer-based UNI model trained on the NCT dataset. Each row corresponds to one network layer. The first column shows activation atlases generated by feature inversion, summarizing aggregated activation patterns across the embedding space. The second column shows tissue-type attribution maps indicating the dominant tissue class associated with each activation

atlas cell. The third column shows t-SNE projections of patch-level feature embeddings extracted from the same layer, colored by tissue class. Class color codes are shown on the right, assigning one of the nine tissue classes: adipose tissue (ADI), background (BACK), debris (DEB), lymphocytes (LYM), mucus (MUC), smooth muscle (MUS), normal colon mucosa (NORM), cancer-associated stroma (STR), and colorectal adenocarcinoma epithelium (TUM).

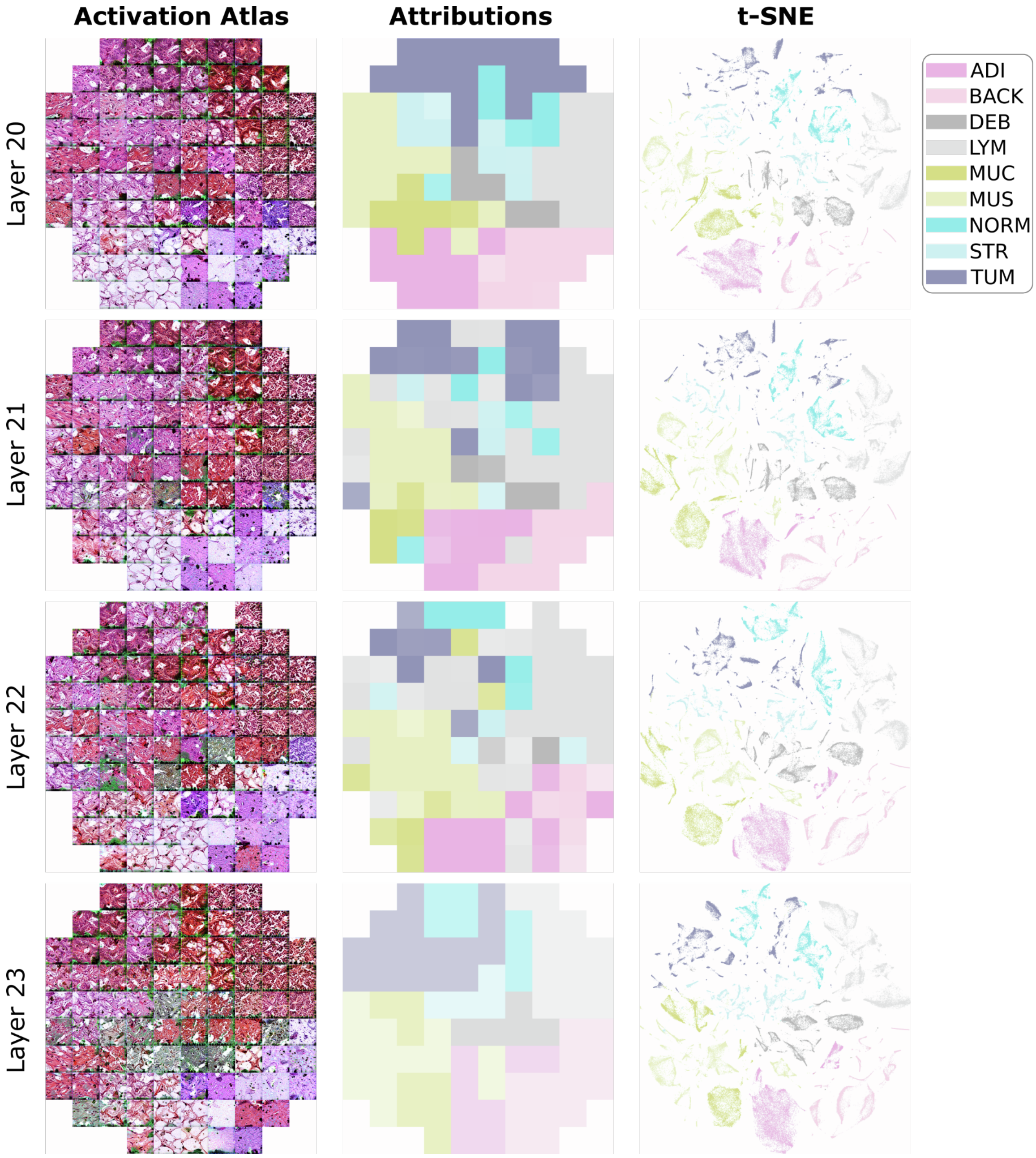

Figure S5 | Activation atlases, class attributions, and embedding projections for layers 20–23 in the National Center for Tumor Diseases (NCT) dataset. Related to Figure 6.
Activation atlases, class attribution maps, and two-dimensional t-distributed stochastic neighbor embedding (t-SNE) projections derived from layers 20–23 (of a total of 24 layers) of the transformer-based UNI model trained on the NCT dataset. Each row corresponds to one network layer. The first column shows activation atlases generated by feature inversion, summarizing aggregated activation patterns across the embedding space. The second column shows tissue-type attribution maps indicating the dominant tissue class associated with each activation atlas cell. The third column shows t-SNE projections of patch-level feature embeddings extracted from the same layer, colored by tissue class. Class color codes are shown on the right, assigning one of the nine tissue classes: adipose tissue (ADI), background (BACK), debris (DEB), lymphocytes (LYM), mucus (MUC), smooth muscle (MUS), normal colon mucosa (NORM), cancer-associated stroma (STR), and colorectal adenocarcinoma epithelium (TUM).

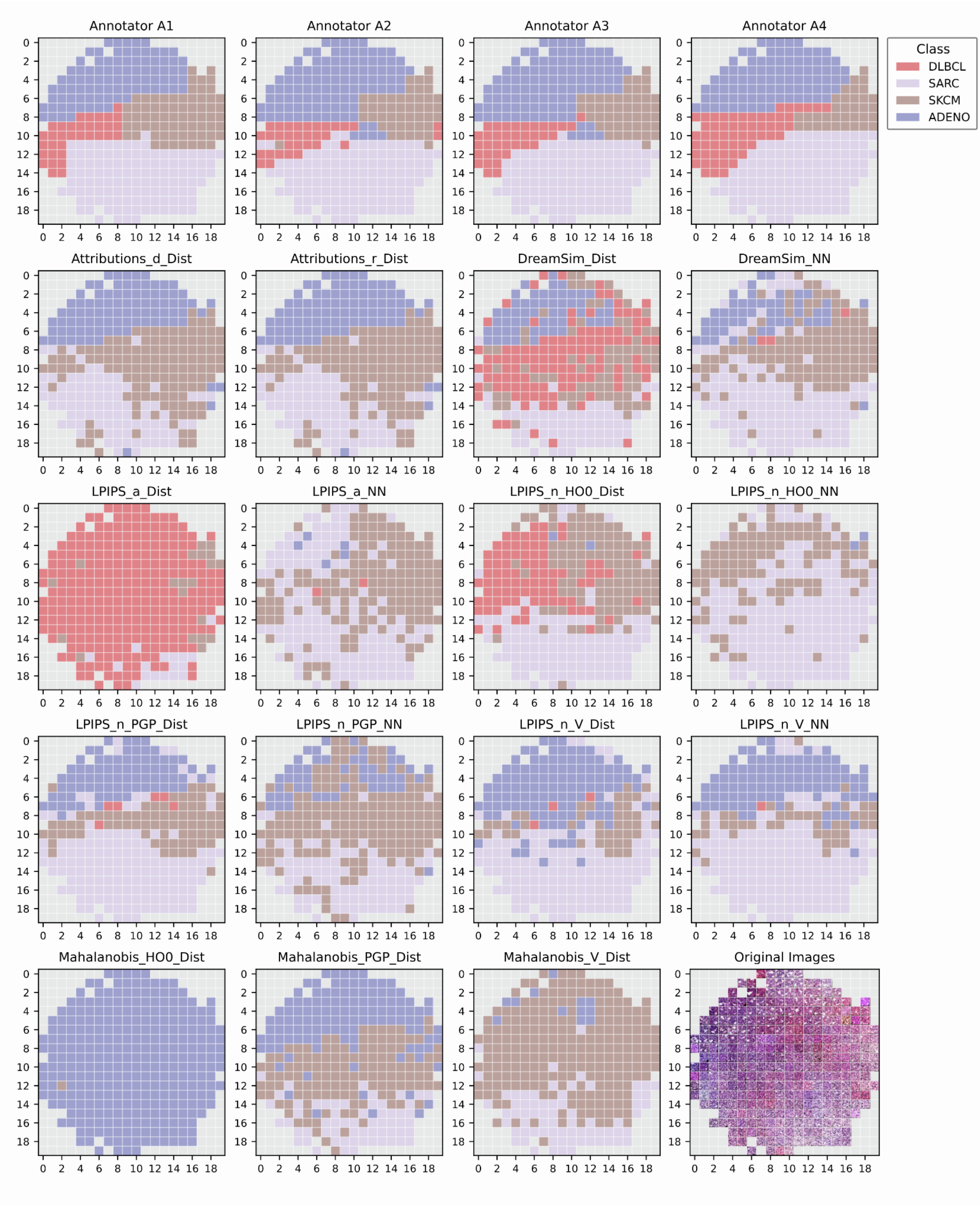

Figure S6 | Label maps for activation atlas cells in The Cancer Genome Atlas dataset (TCGA-5-adeno). Related to Figure 6.

Cell-wise label maps for an activation atlas derived from the TCGA adenocarcinoma-level classification task (TCGA-5-adeno). Each grid shows the same activation atlas layout, in which each cell corresponds to an aggregated region in the reduced activation embedding space. Cell colors indicate the class label assigned to each activation atlas cell.

Top row: Independent annotations by four pathologists (Annotators A1–A4), assigning one of the five TCGA cancer classes: adenocarcinoma (ADENO), lymphoid neoplasm diffuse large B-cell lymphoma (DLBCL), sarcoma (SARC), skin cutaneous melanoma (SKCM), and lung squamous cell carcinoma (LUSC).

Middle rows: Corresponding atlas label maps obtained using quantitative surrogate assignment methods. Attribution-based methods assign labels based on class attributions aggregated over atlas cells, using either distance-based aggregation (_Dist; assignment based on minimal attribution distance) or nearest-neighbor-based aggregation (_NN; assignment based on the closest individual activations). Perceptual similarity-based methods assign each atlas cell to the closest class using either DreamSim or Learned Perceptual Image Patch Similarity (LPIPS), again using distance-based (_Dist) or nearest-neighbor (_NN) strategies. LPIPS variants differ by the feature extractor used, indicated by suffixes _a (AlexNet), _H00 (H-optimus-0), _PGP (Prov-GigaPath), and _V (vision transformer, UNI). Mahalanobis distance-based methods assign labels based on class-conditional feature distributions computed in the corresponding representation spaces.
Bottom right: Generated image patches associated with each activation atlas cell, illustrating the underlying morphological content contributing to the aggregated activations.

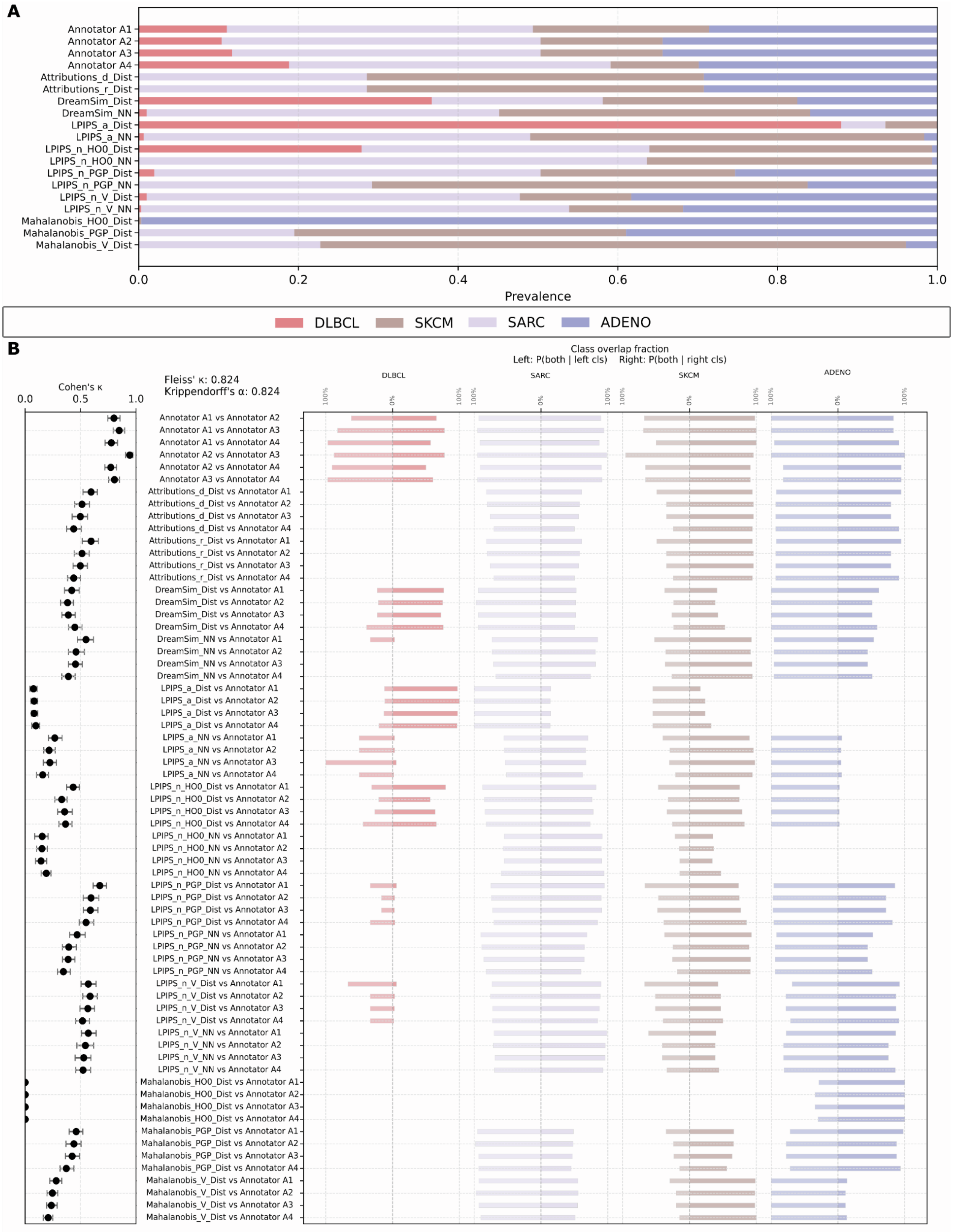

Figure S7 | Agreement and class coverage for activation atlas annotations in The Cancer Genome Atlas (TCGA-5-adeno) cancer task. Related to Figures 5 & 7.

Agreement analysis and class coverage for activation atlas cell annotations derived from the TCGA-5 adenocarcinoma-level classification task.

A. Class coverage. Stacked bar plots showing the distribution of class assignments across activation atlas cells for each pathologist (Annotators A1–A4) and for each metric-based label assignment method. Bars indicate the

proportion of atlas cells assigned to each TCGA-5 cancer class: adenocarcinoma (ADENO), lymphoid neoplasm diffuse large B-cell lymphoma (DLBCL), sarcoma (SARC), and skin cutaneous melanoma (SKCM).
B. Agreement metrics and class-wise overlap. Pairwise agreement between annotators and between annotators and metric-based assignments, computed excluding uncertain labels to ensure comparability of categorical agreement. Left: Cohen's κ values summarizing pairwise agreement, with points indicating mean κ across activation atlas cells and horizontal error bars denoting uncertainty estimated by bootstrap resampling. Overall inter-annotator agreement is summarized by Fleiss' κ and Krippendorff's α (top). Right: Class-wise overlap fractions indicating, for each cancer class, the fraction of activation atlas cells that received the same label from both compared sources. Bars on the left show, for each reference class, how often atlas cells with that reference label are assigned the same class by the comparator. Bars on the right show, for each assigned class, how often the assigned atlas cells originate from the corresponding reference class. Together, these measures capture asymmetric agreement and class-specific ambiguity across expert annotations and quantitative surrogate metrics.

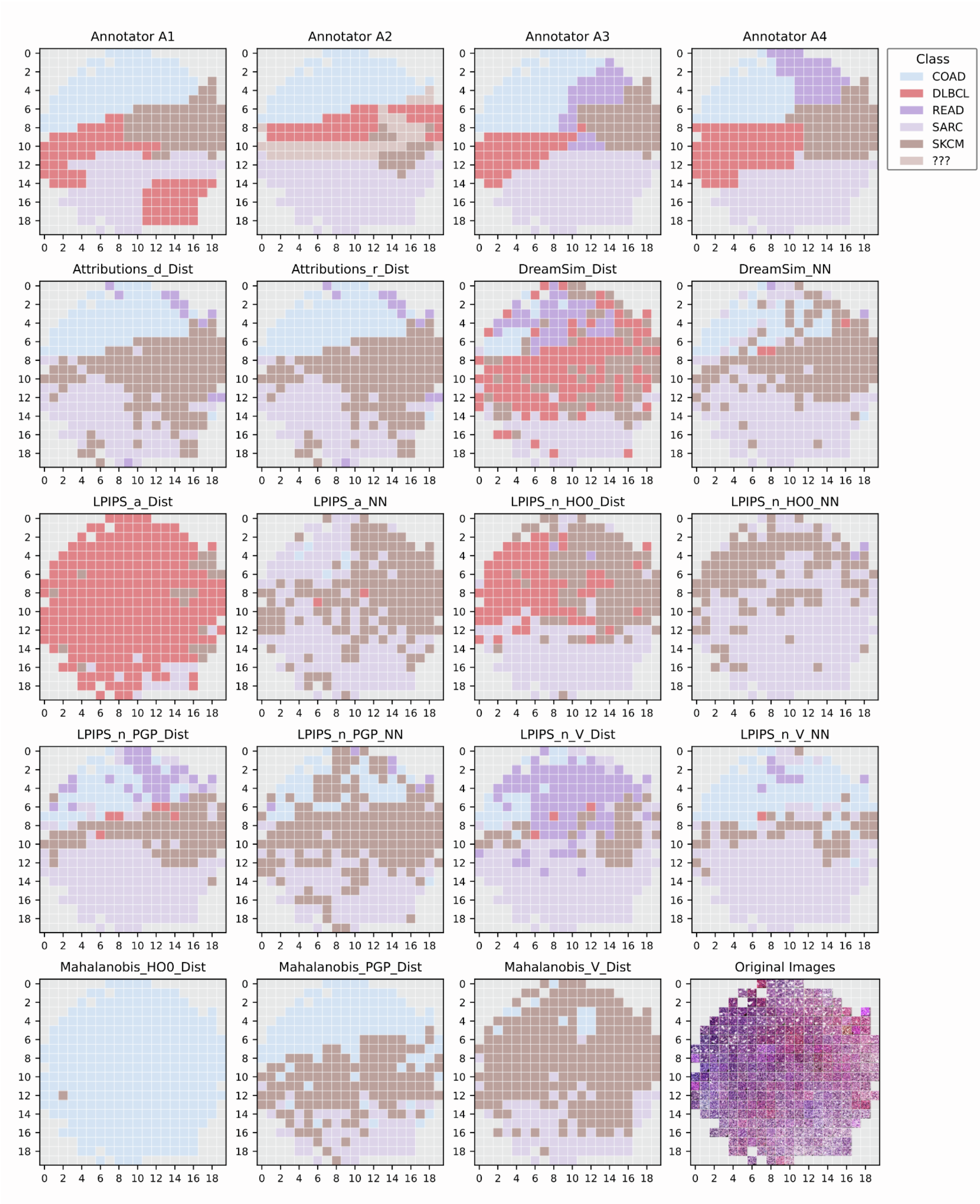

Figure S8 | Label maps for activation atlas cells in The Cancer Genome Atlas dataset (TCGA-5-subclass). Related to Figure 6.

Cell-wise label maps for an activation atlas derived from the TCGA-5 subclass-level classification task. Each grid shows the same activation atlas layout, in which each cell corresponds to an aggregated region in the reduced activation embedding space. Cell colors indicate the class label assigned to each activation atlas cell. Top row: Independent annotations by four pathologists (Annotators A1–A4), assigning one of the five TCGA cancer subclasses: colon adenocarcinoma (COAD), lymphoid neoplasm diffuse large B-cell lymphoma (DLBCL), rectum adenocarcinoma (READ), sarcoma (SARC), and skin cutaneous melanoma (SKCM), or an explicit label for uncertainty (“???”).

Middle rows: Corresponding atlas label maps obtained using quantitative surrogate assignment methods. Attribution-based methods assign labels based on class attributions aggregated over atlas cells, using either distance-based aggregation (_Dist; assignment based on minimal attribution distance) or nearest-neighbor-based aggregation (_NN; assignment based on the closest individual activations). Perceptual similarity-based methods assign each atlas cell to the closest class using either DreamSim or Learned Perceptual Image Patch Similarity (LPIPS), again using distance-based (_Dist) or nearest-neighbor (_NN) strategies. LPIPS variants differ by the feature extractor used, indicated by suffixes _a (AlexNet), _H00 (H-optimus-0), _PGP (Prov-GigaPath), and _V (vision transformer, UNI). Mahalanobis distance-based methods assign labels based on class-conditional feature distributions computed in the corresponding representation spaces.
Bottom right: Generated image patches associated with each activation atlas cell, illustrating the underlying morphological content contributing to the aggregated activations.

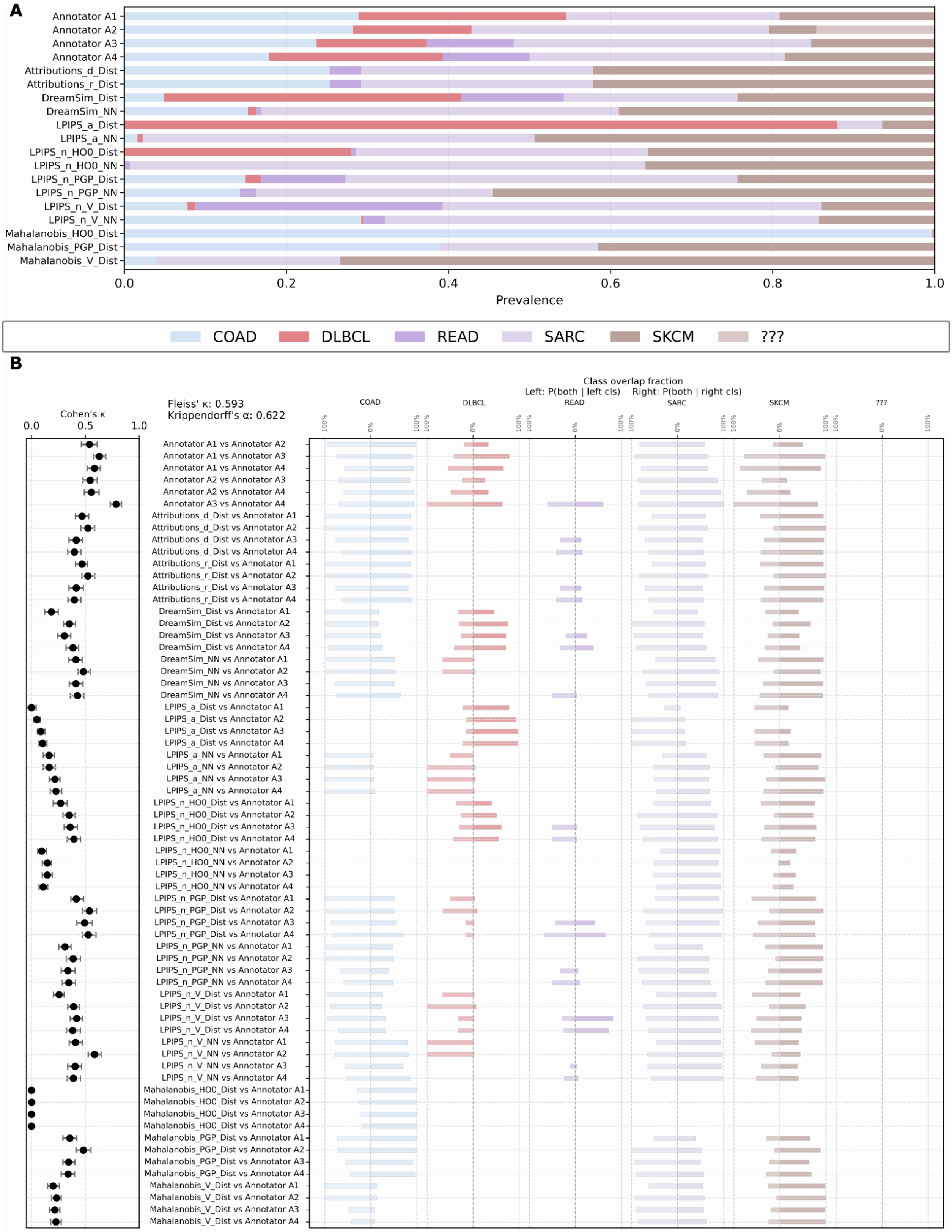

Figure S9 | Agreement and class coverage for activation atlas annotations in The Cancer Genome Atlas (TCGA-5 subclass) cancer task. Related to Figure 5.

Agreement analysis and class coverage for activation atlas cell annotations derived from the TCGA-5 subclass-level classification task.

A. Class coverage. Stacked bar plots showing the distribution of class assignments across activation atlas cells for each pathologist (Annotators A1–A4) and for each metric-based label assignment method. Bars indicate the proportion of atlas cells assigned to each TCGA-5 cancer subclass: colon adenocarcinoma (COAD), lymphoid

neoplasm diffuse large B-cell lymphoma (DLBCL), rectum adenocarcinoma (READ), sarcoma (SARC), and skin cutaneous melanoma (SKCM). Where present, uncertain annotations (“???”) are included as an explicit category to reflect overall class coverage.

B. Agreement metrics and class-wise overlap. Pairwise agreement between annotators and between annotators and metric-based assignments, computed excluding uncertain labels (“???”) to ensure comparability of categorical agreement. Left: Cohen’s κ values summarizing pairwise agreement, with points indicating mean κ across activation atlas cells and horizontal error bars denoting uncertainty estimated by bootstrap resampling. Overall inter-annotator agreement is summarized by Fleiss’ κ and Krippendorff’s α (top). Right: Class-wise overlap fractions indicating, for each cancer subclass, the fraction of activation atlas cells that received the same label from both compared sources. Bars on the left show, for each reference subclass, how often atlas cells with that reference label are assigned the same subclass by the comparator. Bars on the right show, for each assigned subclass, how often the assigned atlas cells originate from the corresponding reference subclass. Together, these measures capture asymmetric agreement and subclass-specific ambiguity across expert annotations and quantitative surrogate metrics.

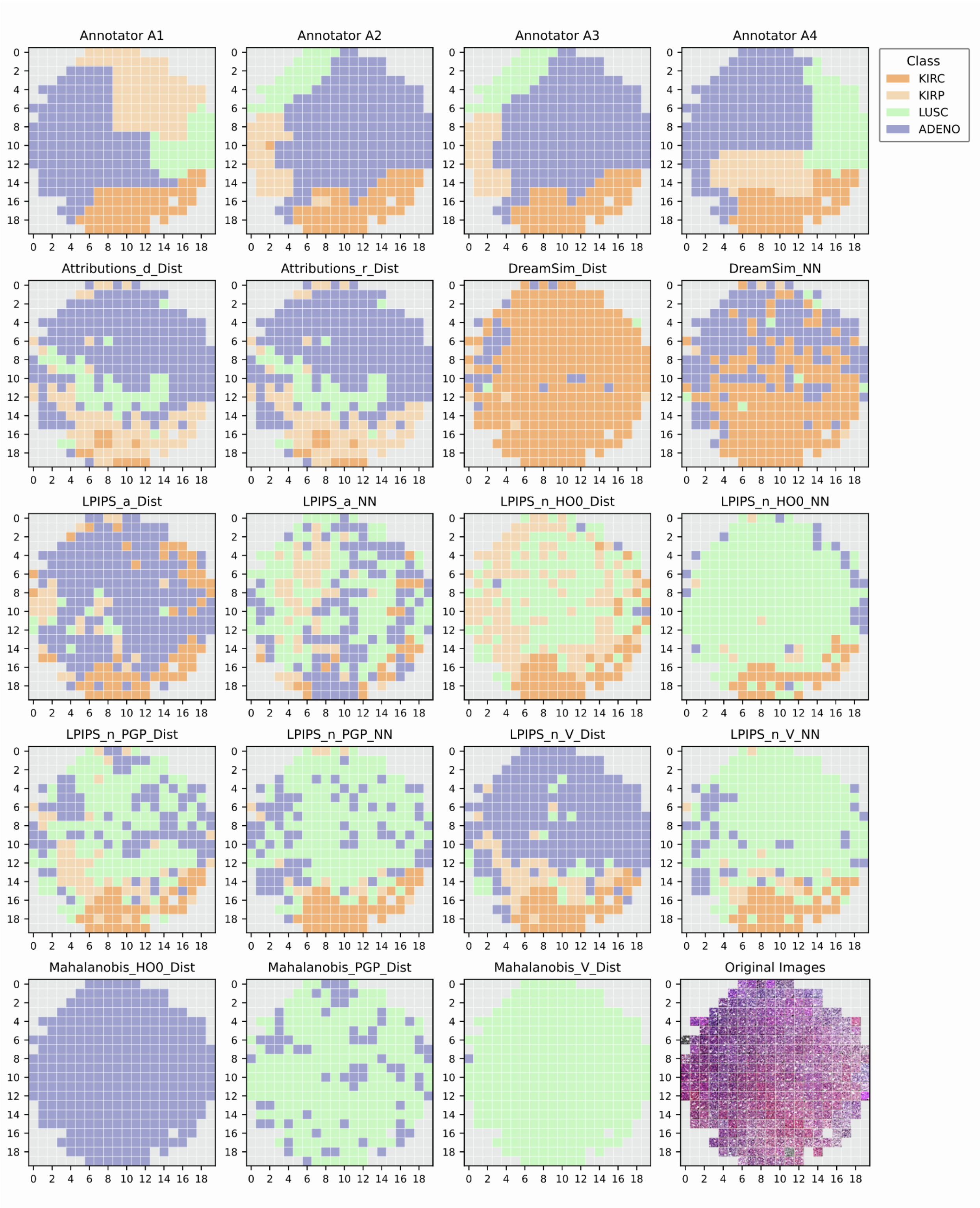

Figure S10 | Label maps for activation atlas cells in The Cancer Genome Atlas dataset (TCGA-8-adeno). Related to Figure 6.

Cell-wise label maps for an activation atlas derived from the TCGA-8 adenocarcinoma-level classification task. Each grid shows the same activation atlas layout, in which each cell corresponds to an aggregated region in the reduced activation embedding space. Cell colors indicate the class label assigned to each activation atlas cell.

Top row: Independent annotations by four pathologists (Annotators A1–A4), assigning one of the four TCGA cancer classes: adenocarcinoma (ADENO), kidney renal clear cell carcinoma (KIRC), kidney renal papillary cell carcinoma (KIRP), and lung squamous cell carcinoma (LUSC).

Middle rows: Corresponding atlas label maps obtained using quantitative surrogate assignment methods. Attribution-based methods assign labels based on class attributions aggregated over atlas cells, using either distance-

based aggregation (_Dist; assignment based on minimal attribution distance) or nearest-neighbor-based aggregation (_NN; assignment based on the closest individual activations). Perceptual similarity-based methods assign each atlas cell to the closest class using either DreamSim or Learned Perceptual Image Patch Similarity (LPIPS), again using distance-based (_Dist) or nearest-neighbor (_NN) strategies. LPIPS variants differ by the feature extractor used, indicated by suffixes _a (AlexNet), _H00 (H-optimus-0), _PGP (Prov-GigaPath), and _V (vision transformer, UNI). Mahalanobis distance-based methods assign labels based on class-conditional feature distributions computed in the corresponding representation spaces.
Bottom right: Generated image patches associated with each activation atlas cell, illustrating the underlying morphological content contributing to the aggregated activations.

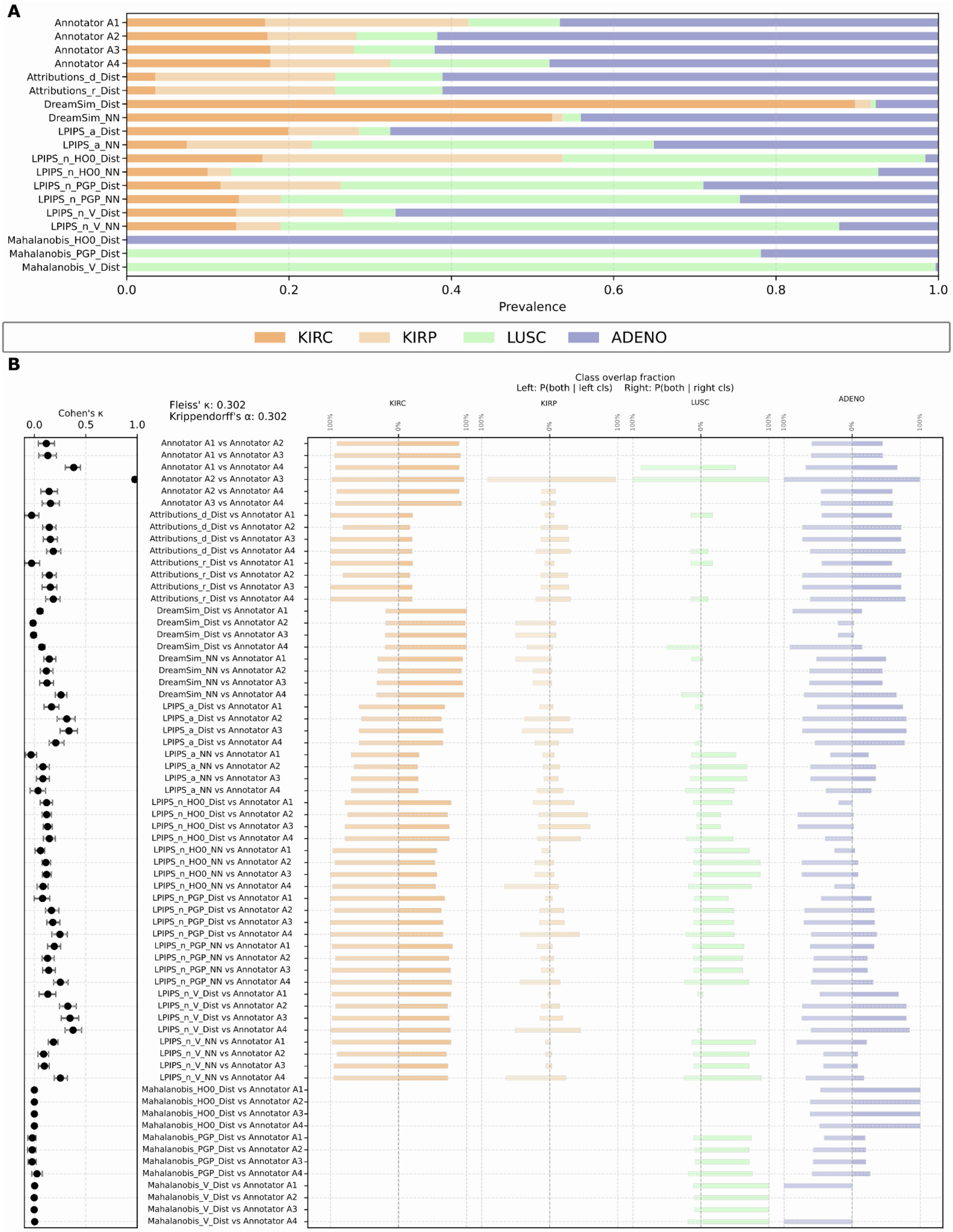

Figure S11 | Agreement and class coverage for activation atlas annotations in The Cancer Genome Atlas (TCGA-8 adeno) cancer task. Related to Figure 5.

Agreement analysis and class coverage for activation atlas cell annotations derived from the TCGA-8 adenocarcinoma-level classification task.

A. Class coverage. Stacked bar plots showing the distribution of class assignments across activation atlas cells for each pathologist (Annotators A1–A4) and for each metric-based label assignment method. Bars indicate the

proportion of atlas cells assigned to each TCGA-8 cancer class: kidney renal clear cell carcinoma (KIRC), kidney renal papillary cell carcinoma (KIRP), lung squamous cell carcinoma (LUSC), and adenocarcinoma (ADENO).

B. Agreement metrics and class-wise overlap. Pairwise agreement between annotators and between annotators and metric-based assignments, computed excluding uncertain labels to ensure comparability of categorical agreement. Left: Cohen's κ values summarizing pairwise agreement, with points indicating mean κ across activation atlas cells and horizontal error bars denoting uncertainty estimated by bootstrap resampling. Overall inter-annotator agreement is summarized by Fleiss' κ and Krippendorff's α (top). Right: Class-wise overlap fractions indicating, for each cancer class, the fraction of activation atlas cells that received the same label from both compared sources. Bars on the left show, for each reference class, how often atlas cells with that reference label are assigned the same class by the comparator. Bars on the right show, for each assigned class, how often the assigned atlas cells originate from the corresponding reference class. Together, these measures capture asymmetric agreement and class-specific ambiguity across expert annotations and quantitative surrogate metrics.

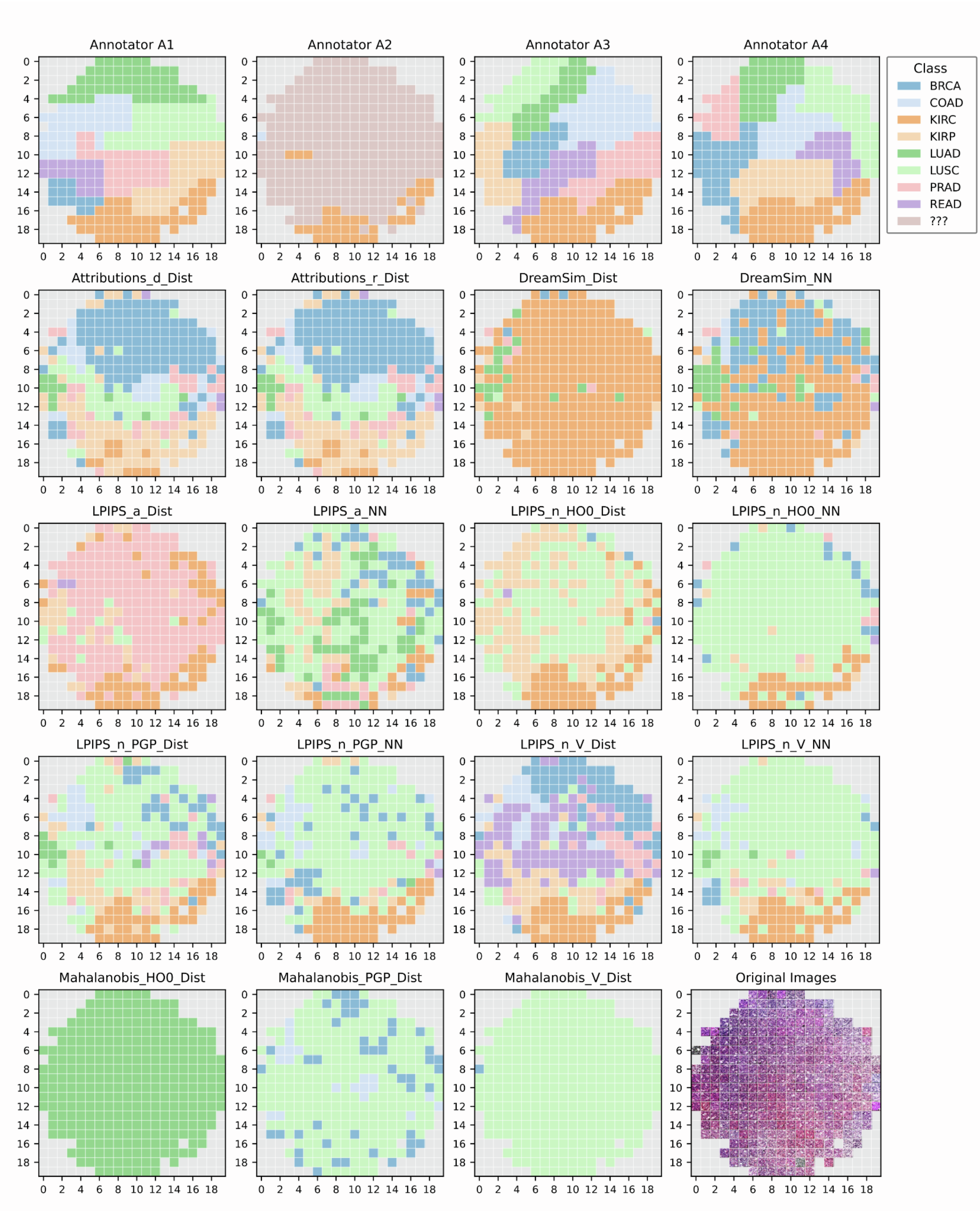

Figure S12 | Label maps for activation atlas cells in The Cancer Genome Atlas dataset (TCGA-8-subclass). Related to Figure 6.

Cell-wise label maps for an activation atlas derived from the TCGA-8 subclass-level classification task. Each grid shows the same activation atlas layout, in which each cell corresponds to an aggregated region in the reduced activation embedding space. Cell colors indicate the class label assigned to each activation atlas cell. Top row: Independent annotations by four pathologists (Annotators A1–A4), assigning one of the eight TCGA cancer subclasses: breast invasive carcinoma (BRCA), colon adenocarcinoma (COAD), kidney renal clear cell carcinoma (KIRC), kidney renal papillary cell carcinoma (KIRP), lung adenocarcinoma (LUAD), lung squamous cell carcinoma (LUSC), prostate adenocarcinoma (PRAD), and rectum adenocarcinoma (READ), or an explicit label for uncertainty ("???").

Middle rows: Corresponding atlas label maps obtained using quantitative surrogate assignment methods. Attribution-based methods assign labels based on class attributions aggregated over atlas cells, using either distance-based aggregation (_Dist; assignment based on minimal attribution distance) or nearest-neighbor-based aggregation (_NN; assignment based on the closest individual activations). Perceptual similarity-based methods assign each atlas cell to the closest class using either DreamSim or Learned Perceptual Image Patch Similarity (LPIPS), again using distance-based (_Dist) or nearest-neighbor (_NN) strategies. LPIPS variants differ by the feature extractor used, indicated by suffixes _a (AlexNet), _H00 (H-optimus-0), _PGP (Prov-GigaPath), and _V (vision transformer, UNI). Mahalanobis distance-based methods assign labels based on class-conditional feature distributions computed in the corresponding representation spaces.
Bottom right: Generated image patches associated with each activation atlas cell, illustrating the underlying morphological content contributing to the aggregated activations.

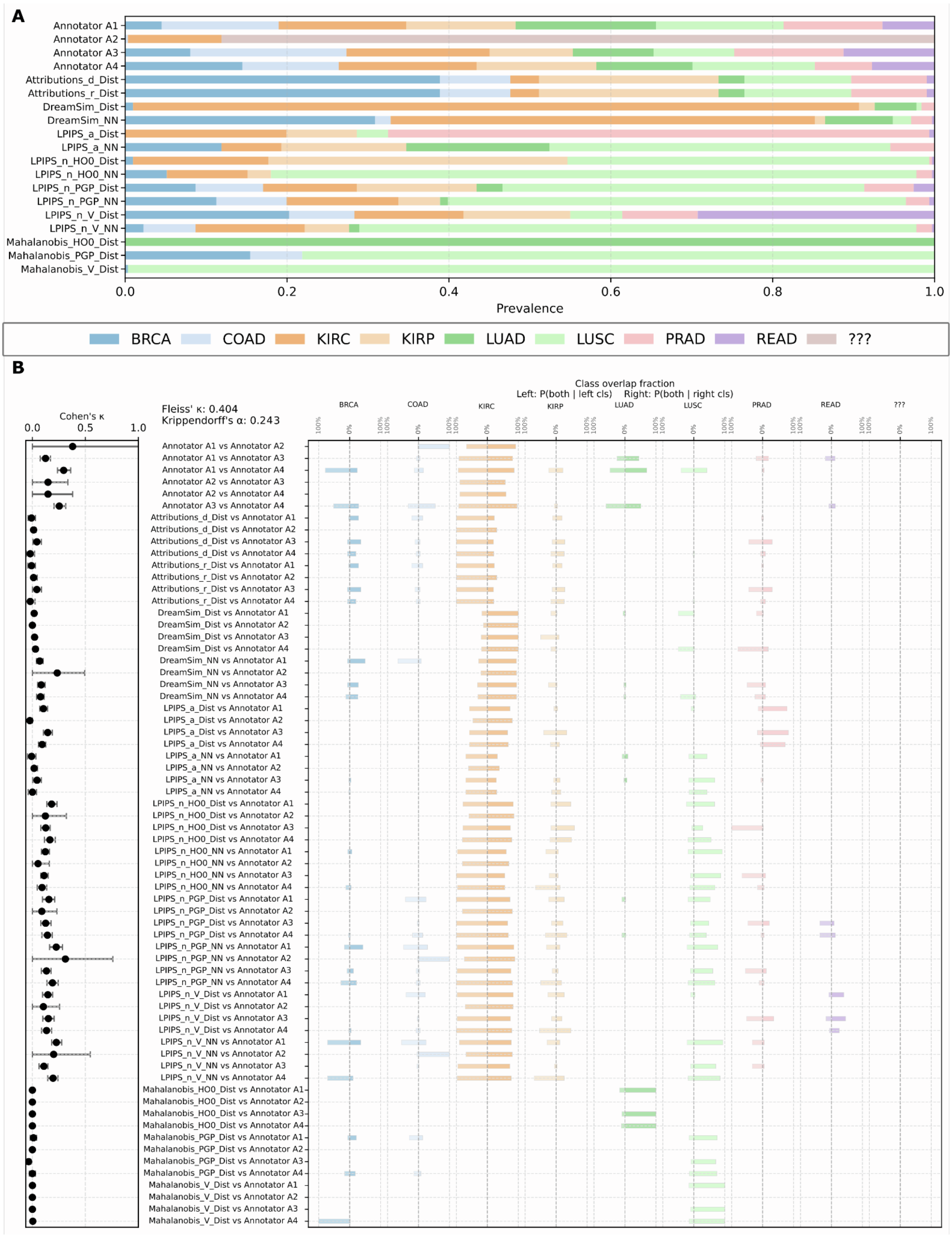

Figure S13 | Agreement and class coverage for activation atlas annotations in The Cancer Genome Atlas (TCGA-8 subclass) cancer task. Related to Figure 5.

Agreement analysis and class coverage for activation atlas cell annotations derived from the TCGA-8 subclass-level classification task.

A. Class coverage. Stacked bar plots showing the distribution of class assignments across activation atlas cells for each pathologist (Annotators A1–A4) and for each metric-based label assignment method. Bars indicate the proportion of atlas cells assigned to each TCGA-8 cancer subclass: breast invasive carcinoma (BRCA), colon adenocarcinoma (COAD), kidney renal clear cell carcinoma (KIRC), kidney renal papillary cell carcinoma

(KIRP), lung adenocarcinoma (LUAD), lung squamous cell carcinoma (LUSC), prostate adenocarcinoma (PRAD), and rectum adenocarcinoma (READ). Where present, uncertain annotations (“???”) are included as an explicit category to reflect overall class coverage.

B. Agreement metrics and class-wise overlap. Pairwise agreement between annotators and between annotators and metric-based assignments, computed excluding uncertain labels (“???”) to ensure comparability of categorical agreement. Left: Cohen’s κ values summarizing pairwise agreement, with points indicating mean κ across activation atlas cells and horizontal error bars denoting uncertainty estimated by bootstrap resampling. Overall inter-annotator agreement is summarized by Fleiss’ κ and Krippendorff’s α (top). Right: Class-wise overlap fractions indicating, for each cancer subclass, the fraction of activation atlas cells that received the same label from both compared sources. Bars on the left show, for each reference subclass, how often atlas cells with that reference label are assigned the same subclass by the comparator. Bars on the right show, for each assigned subclass, how often the assigned atlas cells originate from the corresponding reference subclass. Together, these measures capture asymmetric agreement and subclass-specific ambiguity across expert annotations and quantitative surrogate metrics.

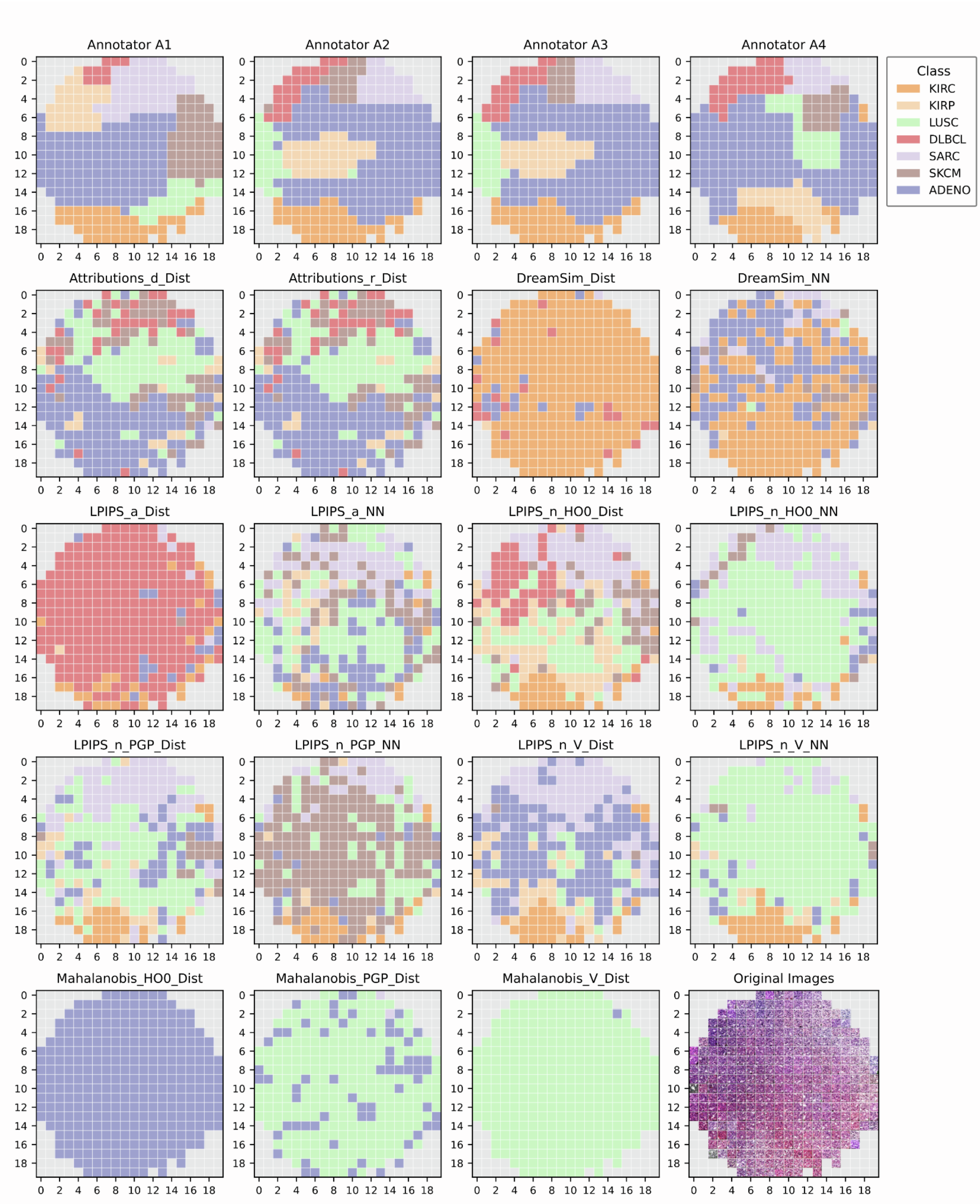

Figure S14 | Label maps for activation atlas cells in The Cancer Genome Atlas dataset (TCGA-11-adeno). Related to Figure 6.

Cell-wise label maps for an activation atlas derived from the TCGA-11 adenocarcinoma-level classification task. Each grid shows the same activation atlas layout, in which each cell corresponds to an aggregated region in the reduced activation embedding space. Cell colors indicate the class label assigned to each activation atlas cell.

Top row: Independent annotations by four pathologists (Annotators A1–A4), assigning one of the six TCGA cancer classes: adenocarcinoma (ADENO), kidney renal clear cell carcinoma (KIRC), kidney renal papillary cell carcinoma (KIRP), lung squamous cell carcinoma (LUSC), lymphoid neoplasm diffuse large B-cell lymphoma (DLBCL), sarcoma (SARC), and skin cutaneous melanoma (SKCM).

Middle rows: Corresponding atlas label maps obtained using quantitative surrogate assignment methods. Attribution-based methods assign labels based on class attributions aggregated over atlas cells, using either distance-based aggregation (_Dist; assignment based on minimal attribution distance) or nearest-neighbor-based aggregation (_NN; assignment based on the closest individual activations). Perceptual similarity-based methods assign each atlas cell to the closest class using either DreamSim or Learned Perceptual Image Patch Similarity (LPIPS), again using distance-based (_Dist) or nearest-neighbor (_NN) strategies. LPIPS variants differ by the feature extractor used, indicated by suffixes _a (AlexNet), _H00 (H-optimus-0), _PGP (Prov-GigaPath), and _V (vision transformer, UNI). Mahalanobis distance-based methods assign labels based on class-conditional feature distributions computed in the corresponding representation spaces.
Bottom right: Generated image patches associated with each activation atlas cell, illustrating the underlying morphological content contributing to the aggregated activations.

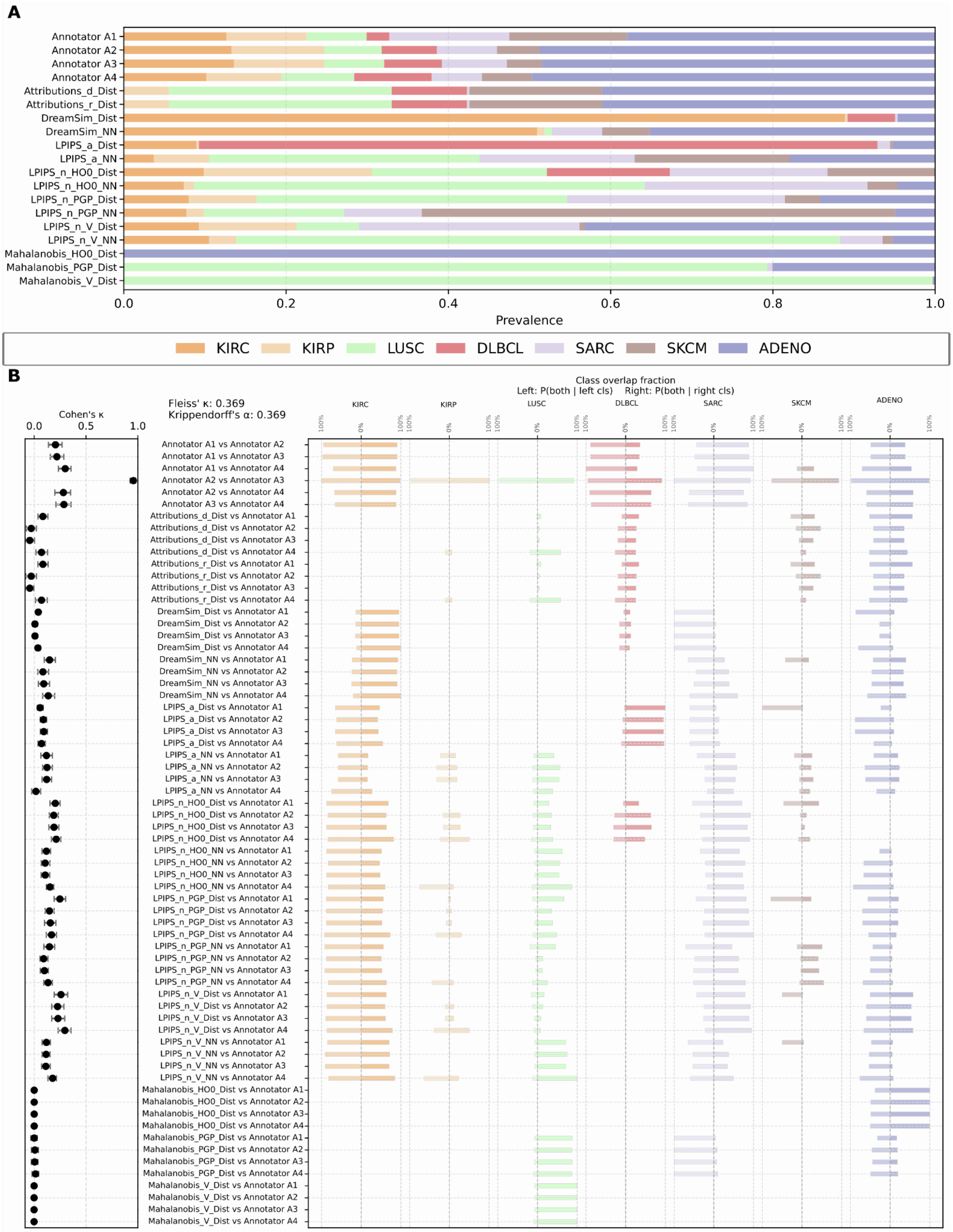

Figure S15 | Agreement and class coverage for activation atlas annotations in The Cancer Genome Atlas (TCGA-11 adeno) cancer task. Related to Figure 5.

Agreement analysis and class coverage for activation atlas cell annotations derived from the TCGA-11 adeno-carcinoma-level classification task.

A. Class coverage. Stacked bar plots showing the distribution of class assignments across activation atlas cells for each pathologist (Annotators A1–A4) and for each metric-based label assignment method. Bars indicate the

proportion of atlas cells assigned to each TCGA-11 cancer class: kidney renal clear cell carcinoma (KIRC), kidney renal papillary cell carcinoma (KIRP), lung squamous cell carcinoma (LUSC), lymphoid neoplasm diffuse large B-cell lymphoma (DLBCL), sarcoma (SARC), skin cutaneous melanoma (SKCM), and adenocarcinoma (ADENO).

B. Agreement metrics and class-wise overlap. Pairwise agreement between annotators and between annotators and metric-based assignments, computed excluding uncertain labels to ensure comparability of categorical agreement. Left: Cohen's κ values summarizing pairwise agreement, with points indicating mean κ across activation atlas cells and horizontal error bars denoting uncertainty estimated by bootstrap resampling. Overall inter-annotator agreement is summarized by Fleiss' κ and Krippendorff's α (top). Right: Class-wise overlap fractions indicating, for each cancer class, the fraction of activation atlas cells that received the same label from both compared sources. Bars on the left show, for each reference class, how often atlas cells with that reference label are assigned the same class by the comparator. Bars on the right show, for each assigned class, how often the assigned atlas cells originate from the corresponding reference class. Together, these measures capture asymmetric agreement and class-specific ambiguity across expert annotations and quantitative surrogate metrics.

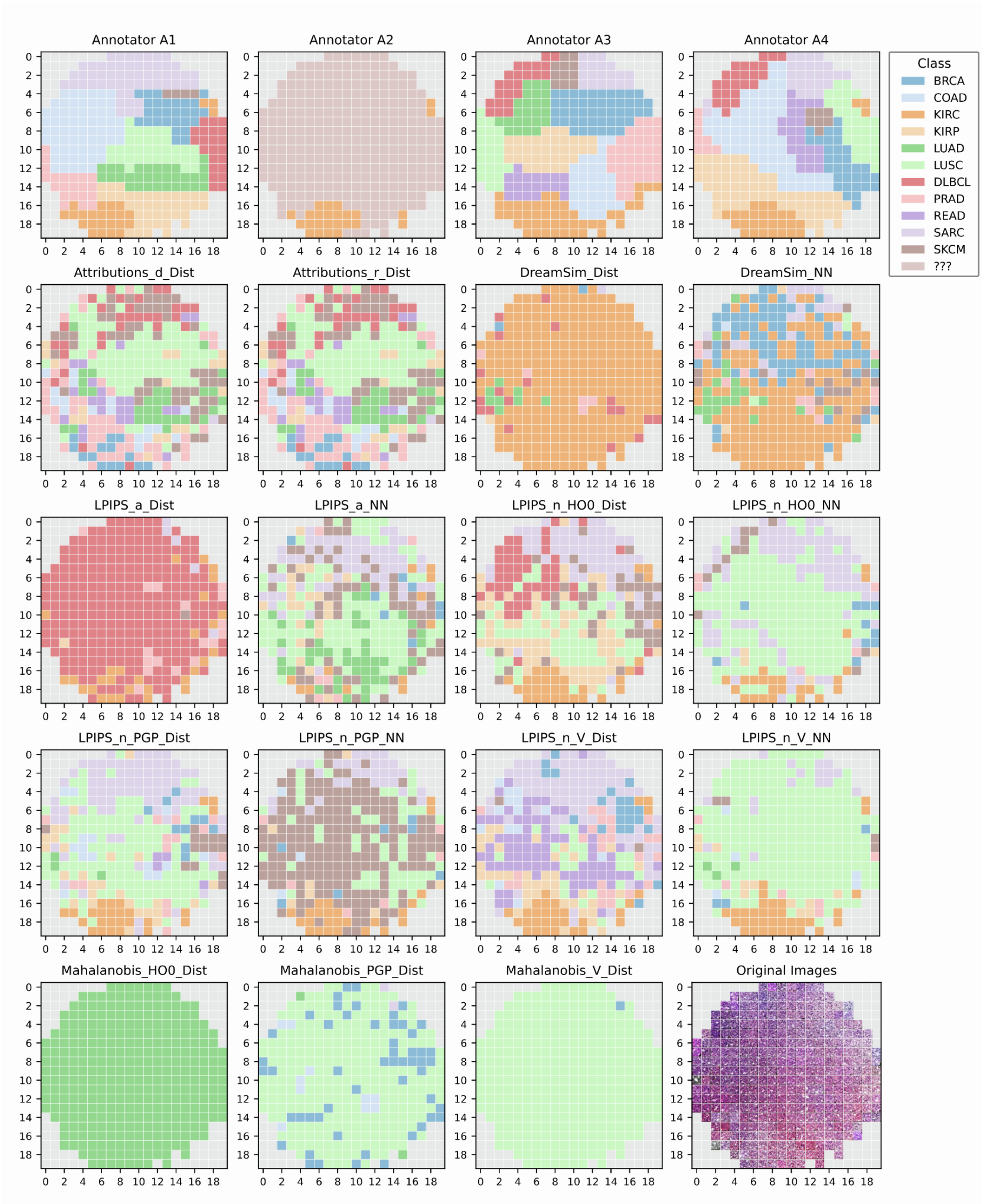

Figure S16 | Label maps for activation atlas cells in The Cancer Genome Atlas dataset (TCGA-11-subclass). Related to Figure 6.
Cell-wise label maps for an activation atlas derived from the TCGA-11 subclass-level classification task. Each grid shows the same activation atlas layout, in which each cell corresponds to an aggregated region in the reduced activation embedding space. Cell colors indicate the class label assigned to each activation atlas cell. Top row: Independent annotations by four pathologists (Annotators A1–A4), assigning one of the eleven TCGA cancer subclasses: breast invasive carcinoma (BRCA), colon adenocarcinoma (COAD), kidney renal clear cell carcinoma (KIRC), kidney renal papillary cell carcinoma (KIRP), lung adenocarcinoma (LUAD), lung squa-

mous cell carcinoma (LUSC), lymphoid neoplasm diffuse large B-cell lymphoma (DLBCL), prostate adenocarcinoma (PRAD), rectum adenocarcinoma (READ), sarcoma (SARC), and skin cutaneous melanoma (SKCM), or an explicit label for uncertainty ("???").
Middle rows: Corresponding atlas label maps obtained using quantitative surrogate assignment methods. Attribution-based methods assign labels based on class attributions aggregated over atlas cells, using either distance-based aggregation (_Dist; assignment based on minimal attribution distance) or nearest-neighbor-based aggregation (_NN; assignment based on the closest individual activations). Perceptual similarity-based methods assign each atlas cell to the closest class using either DreamSim or Learned Perceptual Image Patch Similarity (LPIPS), again using distance-based (_Dist) or nearest-neighbor (_NN) strategies. LPIPS variants differ by the feature extractor used, indicated by suffixes _a (AlexNet), _H00 (H-optimus-0), _PGP (Prov-GigaPath), and _V (vision transformer, UNI). Mahalanobis distance-based methods assign labels based on class-conditional feature distributions computed in the corresponding representation spaces.
Bottom right: Generated image patches associated with each activation atlas cell, illustrating the underlying morphological content contributing to the aggregated activations.

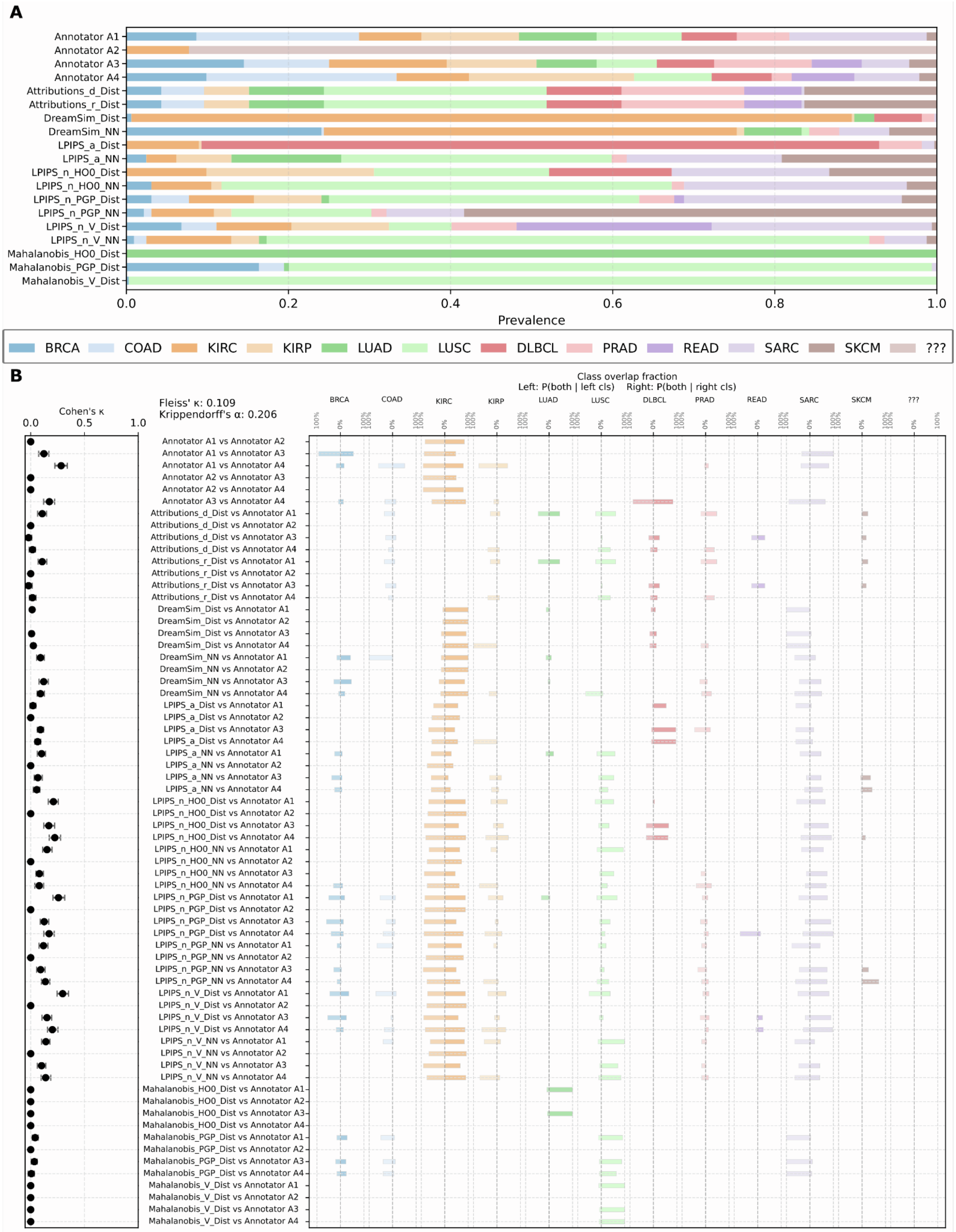

Figure S17 | Agreement and class coverage for activation atlas annotations in The Cancer Genome Atlas (TCGA-11 subclass) cancer task. Related to Figure 5.

Agreement analysis and class coverage for activation atlas cell annotations derived from the TCGA-11 subclass-level classification task.

A. Class coverage. Stacked bar plots showing the distribution of class assignments across activation atlas cells for each pathologist (Annotators A1–A4) and for each metric-based label assignment method. Bars indicate the proportion of atlas cells assigned to each TCGA-11 cancer subclass: breast invasive carcinoma (BRCA), colon

adenocarcinoma (COAD), kidney renal clear cell carcinoma (KIRC), kidney renal papillary cell carcinoma (KIRP), lung adenocarcinoma (LUAD), lung squamous cell carcinoma (LUSC), lymphoid neoplasm diffuse large B-cell lymphoma (DLBCL), prostate adenocarcinoma (PRAD), rectum adenocarcinoma (READ), sarcoma (SARC), and skin cutaneous melanoma (SKCM). Where present, uncertain annotations ("???") are included as an explicit category to reflect overall class coverage.

B. Agreement metrics and class-wise overlap. Pairwise agreement between annotators and between annotators and metric-based assignments, computed excluding uncertain labels ("???") to ensure comparability of categorical agreement. Left: Cohen's κ values summarizing pairwise agreement, with points indicating mean κ across activation atlas cells and horizontal error bars denoting uncertainty estimated by bootstrap resampling. Overall inter-annotator agreement is summarized by Fleiss' κ and Krippendorff's α (top). Right: Class-wise overlap fractions indicating, for each cancer subclass, the fraction of activation atlas cells that received the same label from both compared sources. Bars on the left show, for each reference subclass, how often atlas cells with that reference label are assigned the same subclass by the comparator. Bars on the right show, for each assigned subclass, how often the assigned atlas cells originate from the corresponding reference subclass. Together, these measures capture asymmetric agreement and subclass-specific ambiguity across expert annotations and quantitative surrogate metrics.

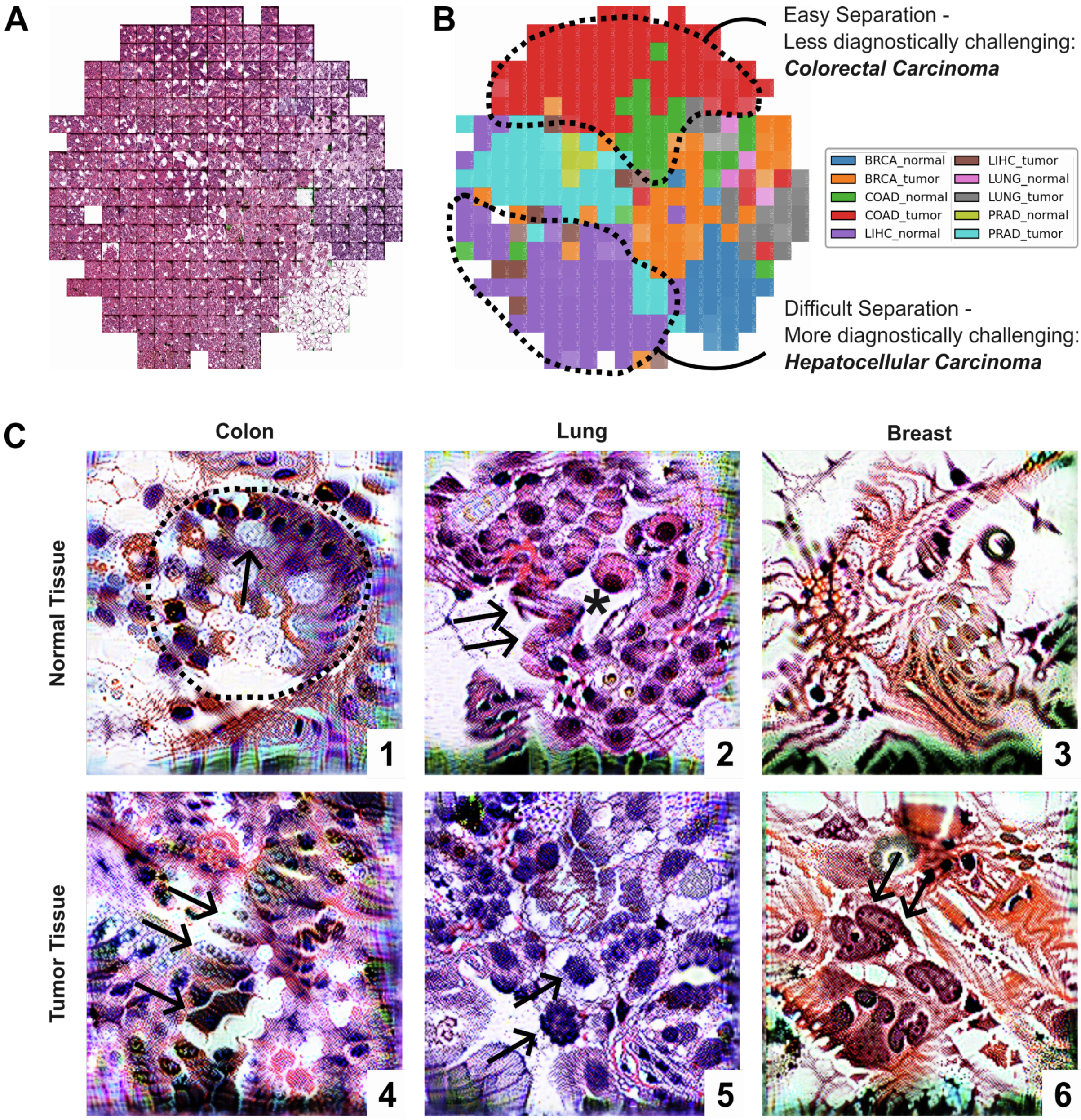

Figure S18 | Exploratory analysis distinguishing between normal adjacent tissue (NAT) and corresponding malignant tissue. Related to the Results.

A–B Activation Atlas of five carcinoma subtypes (colon adenocarcinoma (COAD), breast invasive carcinoma (BRCA), lung adenocarcinoma (LUAD), prostate adenocarcinoma (PRAD), liver hepatocellular carcinoma (LIHC)) and normal adjacent tissue (NAT). In this exploratory atlas, NAT and tumor regions appeared more distinctly separated for COAD than for LIHC.

C CVs show characteristic features of both different healthy tissues and their malignant counterparts upon exploratory, qualitative description. Three representative NAT - carcinoma pairs are shown: normal tissues (**1**–**3**) and their corresponding malignant counterparts (**4**–**6**). **1**: The dashed line surrounds structures highly resembling colonic crypts containing goblet cells (arrows). **4**: COAD displays pleomorphic, hyperchromatic, cigar-shaped nuclei (arrows), reminiscent of colorectal adenoma/adenocarcinoma. **2**: Healthy lung tissue shows structures resembling alveolar septa (arrows) and alveolar macrophages (asterisks). **5**: LUAD shows structures highly reminiscent of (atypical) mitotic figures (arrows). **3**: Healthy breast tissue mostly consists of adipocyte-like shapes, while (**6**) BRCA shows enlarged, pleomorphic, hyperchromatic nuclei with prominent nucleoli (arrows).

Supplementary Tables

Table S1 | Tissue types for the National Center for Tumor Diseases (NCT) dataset[1] and cancer types for The Cancer Genome Atlas (TCGA) dataset[2].

Dataset	No.	Name	Abbreviation
NCT	1	Adipose	ADI
	2	Background	BACK
	3	Debris	DEB
	4	Lymphocytes	LYM
	5	Mucus	MUC
	6	Smooth muscle	MUS
	7	Normal colon mucosa	NORM
	8	Cancer-associated stroma	STR
	9	Colorectal adenocarcinoma epithelium	TUM
	10	Uncertain	???
TCGA	1	Breast invasive carcinoma	BRCA
	2	Colon adenocarcinoma	COAD
	3	Kidney renal clear cell carcinoma	KIRC
	4	Kidney renal papillary cell carcinoma	KIRP
	5	Lung adenocarcinoma	LUAD
	6	Lung squamous cell carcinoma	LUSC
	7	Lymphoid neoplasm diffuse large B-cell lymphoma	DLBCL
	8	Prostate adenocarcinoma	PRAD
	9	Rectum adenocarcinoma	READ
	10	Sarcoma	SARC
	11	Skin cutaneous melanoma	SKCM
	12	Uncertain	???

Table S2 | Statistical comparison of pathologist annotation performance and agreement between real scan images and class visualizations (CVs).

Each row reports one metric for one dataset/task and uncertainty setting across the National Center for Tumor Diseases (NCT) dataset and The Cancer Genome Atlas (TCGA) datasets, corresponding to Figure 5. Scans and CVs denote the values for real scan images and class visualizations, respectively; Δ = metric(scans) – metric(CVs) is shown with its 95% bootstrap confidence interval. For per-annotator metrics, values represent means across the four annotators; Fleiss' κ and Krippendorff's α are group-level statistics. "Uncertain" indicates whether uncertain annotations were retained as a separate category or excluded. Columns with identical values across all 42 comparisons are omitted for clarity: scans exceeded CVs in every comparison, all 95% confidence intervals excluded zero, and all permutation p values reached the Monte Carlo resolution limit after Holm correction (raw $p < 0.0001$; Holm-adjusted $p < 0.005$; all comparisons significant).

Dataset/Task	Uncertain	Metric	Scans	CVs	Δ (scans−CV)	95% CI lower	95% CI upper
NCT	Included	Accuracy	0.8025	0.5401	0.2623	0.1975	0.3241
NCT	Excluded	Accuracy	0.8233	0.5625	0.2608	0.1944	0.3240
TCGA-11	Included	Accuracy	0.4369	0.0732	0.3636	0.3131	0.4141
TCGA-11	Excluded	Accuracy	0.5285	0.0764	0.4521	0.4012	0.5058
TCGA-11-adeno	Included	Accuracy	0.6414	0.2146	0.4268	0.3636	0.4900
TCGA-11-adeno	Excluded	Accuracy	0.6941	0.2135	0.4806	0.4100	0.5496
NCT	Included	F1 Score	0.7832	0.4666	0.3166	0.2372	0.3711
NCT	Excluded	F1 Score	0.8153	0.5226	0.2927	0.2273	0.3585
TCGA-11	Included	F1 Score	0.3854	0.0702	0.3152	0.2621	0.3673
TCGA-11	Excluded	F1 Score	0.4603	0.0660	0.3943	0.3330	0.4470
TCGA-11-adeno	Included	F1 Score	0.4984	0.1243	0.3741	0.3079	0.4496
TCGA-11-adeno	Excluded	F1 Score	0.5679	0.1209	0.4470	0.3599	0.5256
NCT	Included	Sensitivity	0.7840	0.4954	0.2886	0.2120	0.3411
NCT	Excluded	Sensitivity	0.8279	0.5489	0.2790	0.2151	0.3408
TCGA-11	Included	Sensitivity	0.4179	0.0724	0.3455	0.2973	0.3975
TCGA-11	Excluded	Sensitivity	0.4840	0.0762	0.4078	0.3564	0.4629
TCGA-11-adeno	Included	Sensitivity	0.5190	0.1206	0.3984	0.3394	0.4736
TCGA-11-adeno	Excluded	Sensitivity	0.5884	0.1194	0.4690	0.3922	0.5477
NCT	Included	Specificity	0.9780	0.9485	0.0296	0.0223	0.0365
NCT	Excluded	Specificity	0.9780	0.9450	0.0330	0.0247	0.0409
TCGA-11	Included	Specificity	0.9488	0.9156	0.0332	0.0286	0.0379
TCGA-11	Excluded	Specificity	0.9527	0.9074	0.0454	0.0402	0.0508
TCGA-11-adeno	Included	Specificity	0.9435	0.8775	0.0660	0.0559	0.0762
TCGA-11-adeno	Excluded	Specificity	0.9440	0.8588	0.0853	0.0725	0.0978
NCT	Included	Cohen's Kappa vs GT	0.7784	0.4834	0.2951	0.2222	0.3641
NCT	Excluded	Cohen's Kappa vs GT	0.8007	0.5053	0.2954	0.2206	0.3667
TCGA-11	Included	Cohen's Kappa vs GT	0.3841	-0.0124	0.3965	0.3417	0.4524
TCGA-11	Excluded	Cohen's Kappa vs GT	0.4775	-0.0185	0.4959	0.4392	0.5554
TCGA-11-adeno	Included	Cohen's Kappa vs GT	0.5241	0.0091	0.5149	0.4406	0.5908
TCGA-11-adeno	Excluded	Cohen's Kappa vs GT	0.5858	-0.0007	0.5865	0.5034	0.6695
NCT	Included	Fleiss' Kappa	0.7550	0.3133	0.4418	0.3708	0.5127
NCT	Excluded	Fleiss' Kappa	0.7966	0.3315	0.4652	0.3955	0.5347
TCGA-11	Included	Fleiss' Kappa	0.3528	0.0519	0.3009	0.2382	0.3628
TCGA-11	Excluded	Fleiss' Kappa	0.4323	0.0753	0.3570	0.2815	0.4326
TCGA-11-adeno	Included	Fleiss' Kappa	0.5090	0.0674	0.4416	0.3601	0.5265
TCGA-11-adeno	Excluded	Fleiss' Kappa	0.5769	0.0676	0.5093	0.4195	0.6019
NCT	Included	Krippendorff's Alpha	0.7558	0.3154	0.4404	0.3697	0.5111
NCT	Excluded	Krippendorff's Alpha	0.7973	0.3337	0.4636	0.3942	0.5329
TCGA-11	Included	Krippendorff's Alpha	0.3544	0.0543	0.3001	0.2376	0.3619
TCGA-11	Excluded	Krippendorff's Alpha	0.4341	0.0786	0.3556	0.2804	0.4309
TCGA-11-adeno	Included	Krippendorff's Alpha	0.5102	0.0698	0.4404	0.3592	0.5251
TCGA-11-adeno	Excluded	Krippendorff's Alpha	0.5781	0.0702	0.5079	0.4184	0.6003

References

1. Kather, J. N., Halama, N., & Marx, A. (2018). 100,000 histological images of human colorectal cancer and healthy tissue. https://doi.org/10.5281/zenodo.1214456 https://doi.org/10.5281/zenodo.1214456.

2. Komura, D., Kawabe, A., Fukuta, K., Sano, K., Umezaki, T., Koda, H., Suzuki, R., Tominaga, K., Ochi, M., Konishi, H., et al. (2022). Universal encoding of pan-cancer histology by deep texture representations. Cell Rep. *38*, 110424. https://doi.org/10.1016/j.celrep.2022.110424.
